# Psychotropic Drugs Show Anticancer Activity by Disrupting Mitochondrial and Lysosomal Function

**DOI:** 10.3389/fonc.2020.562196

**Published:** 2020-10-19

**Authors:** Marco Varalda, Annamaria Antona, Valentina Bettio, Konkonika Roy, Ajay Vachamaram, Vaibhav Yellenki, Alberto Massarotti, Gianluca Baldanzi, Daniela Capello

**Affiliations:** ^1^Department of Translational Medicine, Centre of Excellence in Aging Sciences, University of Piemonte Orientale, Novara, Italy; ^2^UPO Biobank, University of Piemonte Orientale, Novara, Italy; ^3^Center for Translational Research on Allergic and Autoimmune Diseases (CAAD), University of Piemonte Orientale, Novara, Italy; ^4^Department Pharmaceutical Sciences, University of Piemonte Orientale, Novara, Italy

**Keywords:** lysosomotropism, cationic amphiphilic drugs (CADs), autophagy, psychotropic drug, cancer, repositioning

## Abstract

**Background and Purpose:** Drug repositioning is a promising strategy for discovering new therapeutic strategies for cancer therapy. We investigated psychotropic drugs for their antitumor activity because of several epidemiological studies reporting lower cancer incidence in individuals receiving long term drug treatment.

**Experimental Approach:** We investigated 27 psychotropic drugs for their cytotoxic activity in colorectal carcinoma, glioblastoma and breast cancer cell lines. Consistent with the cationic amphiphilic structure of the most cytotoxic compounds, we investigated their effect on mitochondrial and lysosomal compartments.

**Results:** Penfluridol, ebastine, pimozide and fluoxetine, fluspirilene and nefazodone showed significant cytotoxicity, in the low micromolar range, in all cell lines tested. In MCF7 cells these drugs caused mitochondrial membrane depolarization, increased the acidic vesicular compartments and induced phospholipidosis. Both penfluridol and spiperone induced AMPK activation and autophagy. Neither caspase nor autophagy inhibitors rescued cells from death induced by ebastine, fluoxetine, fluspirilene and nefazodone. Treatment with 3-methyladenine partially rescued cell death induced by pimozide and spiperone, whereas enhanced the cytotoxic activity of penfluridol. Conversely, inhibition of lysosomal cathepsins significantly reduced cell death induced by ebastin, penfluridol, pimozide, spiperone and mildly in fluoxetine treated cells. Lastly, Spiperone cytotoxicity was restricted to colorectal cancer and breast cancer and caused apoptotic cell death in MCF7 cells.

**Conclusions:** The cytotoxicity of psychotropic drugs with cationic amphiphilic structures relied on simultaneous mitochondrial and lysosomal disruption and induction of cell death that not necessarily requires apoptosis. Since dual targeting of lysosomes and mitochondria constitutes a new promising therapeutic approach for cancer, particularly those in which the apoptotic machinery is defective, these data further support their clinical development for cancer therapy.

## Introduction

Cancer represents a major public health problem, with total cure remaining elusive for most cancer types (1, 2). Chemotherapy resistance in patients with recurrent and advanced disease (3) and strong systemic toxicity, especially in elderly (4), have raised concerns over the progress of cancer therapy, making it necessary to change the paradigm in the search for new treatments, more effective and with milder adverse effects. Thus, alternative cell death pathways capable of killing apoptosis- and therapy resistant cancer cells, have gained vast interest among cancer researchers, leading to the identification of autophagy and lysosomal cell death programs as attractive means to circumvent therapy resistance (5–8). Lysosomal activation is common in aggressive cancers, where lysosomes promote disease progression and treatment resistance (9–13). In cancer, cell transformation increases the requirement for new biomass production, and the core function of the lysosomes is to recycle endogenous or exogenous macromolecules to provide energy and metabolic precursors for the synthesis of new cell mass. In response to typical challenges encountered by cancer cells, such as nutrient starvation, growth factor withdrawal, energy depletion, organelle damage, or accumulation of abnormal proteins, autophagy is further enhanced to meet the cellular needs (10, 13). In certain circumstances, however, the prolonged over activation of the autophagosomal/lysosomal pathway can lead to autophagic-dependent cell death a caspase-independent form of programmed cell death (14), that can be evaluated as an alternative cancer treatment modality (15). On the other hand, since many tumors are highly dependent on autophagy for survival and treatment resistance, pharmacological inhibition of lysosomal activity can limit the growth of advanced diseases and improve response to therapy (5, 16). Moreover, the cancer-associated changes in lysosomal composition result in reduced lysosomal membrane stability, thereby sensitizing tumor cells to lysosome-dependent cell death (LDCD) (17). The main feature LDCD is lysosomal membrane permeabilization (LMP) (17, 18) with translocation to the cytoplasm of the lysosomal contents, including cathepsins, which act as the main executors of this cell death modality (19). Mitochondria have a well-recognized role in the production of ATP, metabolic intermediates and also participate in several signaling pathways; accumulating evidence now suggests that mitochondrial bioenergetics, biosynthesis and signaling are required for tumorigenesis. Thus, emerging studies have begun to demonstrate that mitochondrial functions are a potentially fruitful field for cancer therapy (20, 21). Drug repositioning is a strategy for identifying new uses for approved drugs that are outside the scope of the original medical indication (22, 23) and psychotropic medications are promising compounds for cancer treatment. Epidemiological studies have repeatedly reported that individuals who are receiving long term drug treatment with antipsychotics (24, 25), anti-depressant (26–28) or anti-allergic drugs (29) have a lower cancer incidence than the general population, suggesting that these medications might have a direct effect on neoplastic cells. Pre-clinical studies confirmed the direct anti-tumoral activity of these compounds in a wide range of malignancies (30–34). However, despite the large body of experimental evidence, the mechanisms of actions of these compounds in cancer cells remain poorly defined.

In this study we screened a panel of psychotropic compounds for their cytotoxicity in different tumor cell lines to clarify the pharmacological properties underpinning their clinical application for cancer therapy. We identified a group of drugs characterized by cationic amphiphilic properties impairing both mitochondrial and lysosomal function and reducing cancer cells viability at clinically relevant concentrations.

## Methods

### Cell Culture

HCT116, SW620, MCF7, MDA-MB-231, U87 and U251 cell lines were purchased from the American Type Culture Collection (ATCC). HCT116, MCF7, and U251 cells were cultured in Dulbecco's Modified Eagle Medium (DMEM, Gibco; Life Technologies) supplemented with 10% fetal bovine serum (FBS, Euroclone) and 1% antibiotics and antimycotics (Penicillin, Streptomycin, Amphotericin, Sigma). SW620 and MDA-MB-213 cells were cultured in RPMI-1640 (Gibco, Life Technologies) with 10% fetal bovine serum (FBS, Euroclone) and 1% antibiotics and antimycotics (Penicillin, Streptomycin, Amphotericin, Sigma). U87 cells were cultured in Minimum Essential Medium (MEM, Gibco; Life Technologies) with 10% FBS and 1% antibiotics and antimycotics. All the cell lines were maintained in incubator at 37°C with 5% CO_2_.

### Drugs

Psychotropic drugs used in the screening were purchased from Cayman Chemicals, Sigma, TCI Chemicals and Selleck Chemicals. List of drug used: aripiprazole, brexpiprazole, cetirizine, diphenhydramine, droperidol, ebastine, fluoxetine, fluspirilene, haloperidol, iloperidone, ketanserin, metoclopramide, nefazodone, paliperidone, penfluridol, pimozide, pipamperone, R59022, R59949, risperidone, ritanserin, spiperone, trazodone, urapidil, way-100135, and ziprasidone. All drugs were dissolved in DMSO at a 10 mmol/L concentration and stored, in small aliquots at −20°C.

### MTT Viability Assay

For each cell line, 1000 cells/well were plated in a volume of 100 μL in 96 wells plate. Cells were treated with different concentrations of drug (160, 80, 40, 20, and 10 μmol/L) and incubated for 72 h. For each concentration of drug, the same concentration of vehicle (DMSO) was used as control. MTT (thiazolyl blue tetrazolium bromide, Sigma) 0.5 mg/ml was, then, added to each well and incubated for 4 h at 37°C and 5% CO_2_. Crystals were dissolved using 100 μl of acidic isopropanol (4 mmol/L HCl) and the absorbance (570 and 650 nm) was read at the spectrophotometer (Victor, PerkinElmer).

To perform viability assay with biogenic amines 4,000 cells/well from MCF7 and HCT116 were plated in 96 wells plate. Cells were treated with different doses of serotonin, dopamine and histamine (Cayman Chemicals) in DMEM 0% FBS and viability was evaluated after 24- and 48-h treatment by MTT assay.

### Viability Rescue Assay

To perform viability rescue experiments, 1,500 MCF7 cells were plated in 96 wells plate and treated with 10 μmol/L spiperone, nefazodone, fluoxetine, fluspirilene, ebastine, pimozide or 5 μmol/L penfluridol in combination with vehicle alone (DMSO), or with 5 μmol/L carbobenzoxy-valyl-alanyl-aspartyl- [O-methyl]fluoromethylketone (zVAD-fmk, AdipoGen), 2.5 mmol/L 3-methyladenine (3-MA, AdipoGen), 5 mmol/L N-[[(2S,3S)-3-[(propylamino) arbonyl]-2-oxiranyl]carbonyl]-L-isoleucyl-L-proline, methyl ester (CA-074 me, Cayman Chemical), 5 μmol/L cyclosporin A (Cayman Chemical) and 5 μmol/L N-Acetyl-L-cysteine (NAC, Sigma Aldrich). MTT viability assay was performed after 72 h as previously described, except for NAC where, prior to MTT adding, medium was removed and each well was washed with 100 μL of phosphate buffered saline. For biogenic amines viability rescue, 1,500 MCF7 cells were seeded in 96 wells plate and treated with IC_50_ concentration of the following drugs: spiperone, nefazodone, fluoxetine, fluspirilene, ebastine, pimozide, penfluridol in combination with vehicle (DMSO) or 5 μmol/L dopamine, serotonin or histamine. MTT viability assay was performed as described before after 24, 48, and 72 h.

### Apoptosis Assay

Fifty thousand MCF7 cells were plated in 24 wells plate and treated for 48 h with 10 μmol/L fluoxetine, ebastine, pimozide, fluspirilene, spiperone, nefazodone, or 5 μmol/L penfluridol.

Cells were then stained following the manufacturer's instruction (AdipoGen). Briefly, cells were incubated for 10 min at room temperature with annexin binding buffer 1X (10 mmol/L HEPES/NaOH, pH 7.4, 140 mmol/L NaCl, 2.5 mmol/L CaCl2) containing Annexin V-FITC. Lastly, cells were washed and resuspended in annexin binding buffer 1X. Propidium iodide was added to all the samples 5 min before FACS analysis (Attune Nxt, Flow Cytometer, Thermo Fisher Scientific). Data were analyzed with FlowJo^TM^ software (Becton, Dickinson and Company).

### Migration Assay

Migration assay was performed using culture-insert 2 well in μ-dish (ibidi GmbH, Martinsried, Germany) as previously described (35). Briefly 30,000 HCT116 cells and 25,000 MCF7 cells were plated in each side of the insert in 24 wells plate. After 24 h, inserts were removed, and cells were treated with respective psychotropic drugs (5 μmol/L) or DMSO (0.05%) in complete medium. Images were acquired at 0 and 24 h after treatment, with phase contrast microscope and analyzed through ImageJ software (NIH, USA). Data were shown as % of closure rate relative to time 0.

### Vacuolization Assay

MCF7 cells were plated at the concentration of 25,000 cells/well in 48 wells plate and then treated with fluoxetine, ebastine, penfluridol, pimozide, fluspirilene, spiperone, nefazodone at the concentration of 5 μmol/L or rapamycin (10 μmol/L). After 2 h treatment one well from each treatment was treated with bafilomycin A1 (50 nmol/L) or 3-MA (1 mmol/L). Pictures were acquired with a phase contrast microscope 4 and 6 h after treatment, images were analyzed by ImageJ software. Analysis shows the percentage of vacuolization rate for each treatment.

### Mitochondrial Membrane Potential Analysis

Mcf7 cells were plated at the concentration of 20,000 cells/well in 48 wells plate and treated with 5 μmol/L fluoxetine, ebastine, fluspirilene, nefazodone penfluridol, pimozide, spiperone. DMSO 0.05% was used as negative control. After treatment, cells were stained with 10 μg/ml JC-1 dye (Adipogen) in PBS for 30 min in the dark at 37°C. FCCP (Cayman chemicals) was added for 15 min after the staining as positive control. Signals were acquired with a fluorescence microscope (FLoid Cell Imaging Station, Life Technology) and images were analyzed by ImageJ software calculating red/green fluorescence ratio.

### Lysotracker Assay

MCF7 cells were plated at the concentration of 20,000 cells/well in 48 wells plate and treated with 5 μmol/L fluoxetine, ebastine, fluspirilene, nefazodone penfluridol, pimozide, spiperone or 10 μmol/L rapamycin for 16 h. After the treatment, medium was removed and cells were stained with Lysotracker Deep Red (Invitrogen, 50 nmol/L) and Hoechst 33342 (5 μg/ml) for nuclei staining, in the dark at 37°C for 30 min. Signals were acquired with a fluorescence microscope (FLoid Cell Imaging Station, Life Technology). Lysotracker red signal/blue nuclei signal was analyzed by ImageJ software.

### Phospholipidosis Assay

MCF7 cells were plated at the concentration of 20,000 cells/well in 48 wells plate and treated with 5 μmol/L ebastine, fluoxetine, fluspirilene, nefazodone penfluridol, pimozide, spiperone or 10 μmol/L rapamycin and stained with 1X LipidTox green (Thermo Fisher Scientific) for 16 h.

Subsequently, nuclei were stained using Hoechst 33342 (5 μg/ml) and plate was incubated for 30 min in the dark at 37°C. Afterwards, cells were washed with PBS and fixed with paraformaldehyde 4% for 15 min in the dark. Signals were acquired with a fluorescence microscope (FLoid Cell Imaging Station, Life Technology) and images were analyzed by ImageJ software.

### Western Blotting

MCF7 cells were plated at the concentration of 150,000 cells/well in 6 wells plate and treated with 5 μmol/L ebastine, fluoxetine, fluspirilene, nefazodone penfluridol, pimozide, spiperone for 16 h. For experiment of autophagic flux two conditions were carried out for each drug: drug alone and co-treatment of drug and chloroquine 50 μmol/L. For experiment to evaluate LC3B expression upon 3-MA treatment, cells were pre-treated with 3-MA 1 mmol/L for 2 h and then cotreated with spiperone and penfluridol 5 μmol/L for 16 h. After treatments, whole cell lysates were prepared using RIPA lysis buffer (25 mmol/L Hepes pH 8, 135 mmol/L NaCl, 5 mmol/L EDTA, 1 mmol/L EGTA, 1 mmol/L ZnCl2, 50 mmol/L NaF, 1% Nonidet P40, 10% glycerol) with protease inhibitors (AEBSF, aprotinin, bestatin, E-64, EDTA, leupeptin, Sigma-Aldrich) and orthovanadate. Lysates were then kept on a wheel for 20 min at 4°C and after centrifuged at 12,500 g for 15 min. Proteins contained in the samples were collected and quantified using Pierce BCA protein assay kit (Thermo Fisher Scientific). Successively, proteins were denatured at 95°C for 5 min in presence of 2% Sodium Dodecyl Sulfate (SDS), 150 mmol/L dithiothreitol (DTT) and 0.01% bromophenol blue. Electrophoresis of the samples was performed using 6, 8, 10, or 15% polyacrylamide gels and proteins were transferred from the gel to a PolyVinylidene DiFluoride membrane (PVDF, Amersham). Lastly, the membrane was saturated using 3% Bovine Serum Albumin (BSA, Sigma) in TBS/Tween-20 0.1% [Tris Buffered Saline 1X containing Trizma base 50 mmol/L, NaCl 120 mmol/L, 0.1% Polyethylene glycol sorbitan monolaurate (Tween-20)] for 1 h and incubated with primary antibody dissolved in the same buffer with sodium azide 0.01%. Primary antibodies were anti-LC3B (Thermo Scientific), anti-P-P70S6K T389 (Cell Signaling Technology), anti-P70S6K (Cell Signaling Technology), anti-P-S6 S235/236 (Cell Signaling Technology), anti-S6 (Cell Signaling Technology) anti-P-AMPKα T172 (Cell Signaling Technology), anti-AMPK (Cell Signaling Technology), anti-GAPDH (Cell Signaling Technology). The day after, primary antibody was removed and the membrane was washed with TBS-Tween-20 0.1% for 15 min three times and then incubated with horseradish peroxidase conjugated secondary anti-mouse or anti-rabbit antibody (Perkin Elmer Life Science) diluted 1:3000 in TBS-Tween-20 0.1% for 45 min. After washing, reading of the membrane was performed using ECL Western Lightning Chemiluminescence Reagent Plus (Perkin Elmer Life Science) and images acquired with the Chemidoc Touch (Bio-Rad).

### Immunofluorescence Microscopy Analysis

MCF7 cells at the concentration of 50,000 cells/well were seeded onto glass coverslips and treated with 5 μmol/L fluoxetine, ebastine, penfluridol, pimozide, fluspirilene, spiperone, nefazodone for 16 h. After the treatment, cells were washed with PBS and fixed with PFA 4% for 10 min at room temperature and washed with PBS. Then cells were permeabilized incubating with cold HEPES-Triton X-100 (20 mM HEPES pH 7.4, 300 mM sucrose, 50 mM NaCl, 3 mM MgCl_2_, 0.5% Triton X-100) for 5 min at 4°C. Cells were washed with 0.2% PBS-BSA and saturated using 2% PBS-BSA for 15 min before placing primary antibodies.

Antibodies used in these experiments were anti-mTOR (Cell Signaling Technology), anti-Galectin-1 (Santa Cruz Biotechnology), anti-LAMP1 (Santa Cruz Biotechnology). Cells were incubated with primary antibodies for 30 min, then washed, saturated with 2% PBS-BSA and incubated with secondary antibodies conjugated with Alexa Fluor-488, −536 (Invitrogen) and DAPI for 30 min.

After the incubation, glasses were mounted on glass slides using Mowiol (20% Mowiol 4–88, 2.5% DABCO in PBS, pH 7.4). Images were acquired at confocal microscope Leica TCS SP8 or fluorescence microscope DM5500B (Leica) and analyzed using ImageJ software.

### Compounds Chemical Analysis

The properties of the compounds (LogP and basic pKa) were investigated using ACD/ LAB software. As reported in the publication of Muehlbacher (36) there is not a clear CADs classification based on chemical properties. We decided to apply the same parameters based on LogP and pKa applied in the Muehlbacher's manuscript. In particular, compounds were considered CADs when LogP > 3, for the amphiphilic characteristics, and a PKa > 7.4 for the cationic characteristics.

### Statistical Analysis

Prism 8.0 software was used for statistical analysis (GraphPad software Inc., San Diego, CA). In viability assays, IC_50_ was determined using a variable slope model referring to the values obtained during the assay; a semi-logarithmic dose-response curve was created.

Statistical significance was analyzed using Student's *t*-test with *p* < 0.05 as the criterion of significance when two groups were compared. Analysis of contingency tables were performed using Prism 8.0 software (GraphPad software Inc., San Diego, CA) and statistical significance was evaluated using Fisher exact test with *p* < 0.05.

## Results

### The Antitumoral Activity of Psychotropic Drugs Transcends the Conventional Therapeutic Classes and Tumor Type

**Figure 1:**
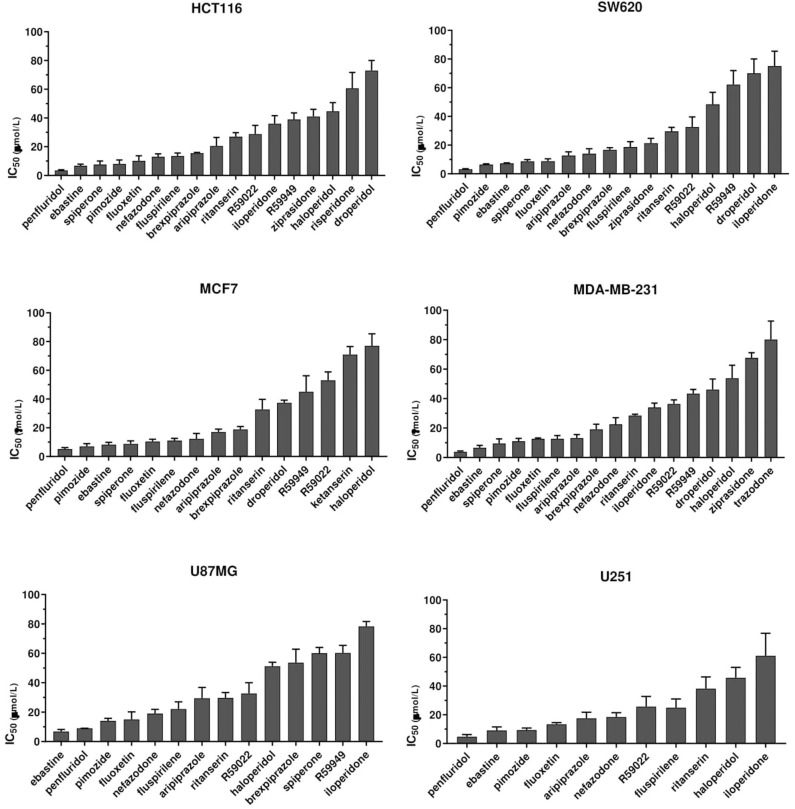
Anticancer activity of psychotropic drugs. Two CRC (HCT116 and SW620), 2 BC (MCF7 and MDA-MB-231) and 2 GB (U87MG and U251) cell lines were treated for 72 h with scalar doses of drugs ranging from 10 to 160 μmol/L. The screened drugs included antipsychotics, antidepressant, antihistamines, and three compounds used in scientific research with reported serotonin receptors antagonistic activity (R59949, R59022; WAY-100135). Viabilities were assessed by MTT assay. Data are presented as mean IC_50_ ± standard error of the mean (SEM) from three-five independent experiments, each performed in quadruplicate. IC_50_, drug concentration reducing by 50% viability compared to control. Histograms show drugs with IC_50_ < 100 μmol/L.

To identify compounds with potential, clinically relevant, anticancer activity we first assessed their effect on six different tumor types represented by two colorectal cancer (CRC; HCT116 and SW620), two breast cancer (BC, MCF7, and MDA-MB-231) and two glioblastoma (GB; U87MG and U251MG) cell lines. Cells were treated for 72 h with scalar doses of drugs ranging from 10 to 160 μmol/L. The screened drugs (*N* = 26) were represented by antipsychotics (*n* = 14), antidepressant (*n* = 2), antihistamines (*n* = 3) and three compounds used in scientific research with reported serotonin receptors antagonistic activity (Figure 1, Supplementary Figure 1, Supplementary Table 1). For drugs that induced more than 50% cell viability reduction at a concentration lower than 100 μmol/L, in a dose-dependent manner, the IC_50_ values were calculated (Supplementary Figure 2, Supplementary Table 1).

The most effective drugs in all cell lines tested belonged to all three pharmacological classes investigated (antipsychotics, antidepressants, and antihistamines) (Figure 1, Supplementary Figures 1, 2, Supplementary Table 1). The six most potent drugs induced more than 50% cell viability reduction at a concentration lower that 10 μmol/L (penfluridol, ebastine), 15 μmol/L (pimozide and fluoxetine) or 25 μmol/L (fluspirilene and nefazodone) in all cell lines tested; spiperone and brexpiprazole proved to be highly effective in both CRC and BC (with IC_50_ < 10 μmol/L and 10 < IC_50_ < 20 μmol/L, respectively) whereas their cytotoxicity was negligible in GB. A tendency for the diphenylbutylpiperidines pimozide, fluspirilene and penfluridol to be more effective in BC and CRC than in GB was also observed (Supplementary Table 1). Aripiprazole and ritanserin demonstrated a moderate cytotoxicity, whereas droperidol, haloperidol and iloperidone showed a weak effect only in a fraction of cell lines. Notably, in the lower range of concentrations, some compounds induced a moderate increase in cell viability reflecting cell proliferation: haloperidol in all cell lines tested; ritanserin and the two structurally related compounds R59022 and R5949 in CRC cell lines only, whereas iloperidone in MCF7 and U87MG cell lines (Supplementary Figure 1).

Eight compounds, represented by the antihistamines cetirizine and diphenhydramine, the antipsychotics paliperidone, pipamperone and risperidone, the antihypertensives ketanserin and urapidil, and the antiemetic metoclopramide showed no cytotoxicity, or caused a reduction of at least 50% of cell viability only at very high concentrations (>60 μmol/L) (Supplementary Figure 1, Supplementary Table 1). A few of these drugs i.e., urapidil, cetirizine, diphenhydramine and metoclopramide even induced cell growth in one or more cell lines tested (Supplementary Figure 1). These results clearly suggest that the cytotoxic effect of these compounds in the micromolar range is not associated with their conventional pharmacological properties and clinical use.

### Cytotoxicity of Psychotropic Drugs Is Not Mediated by Biogenic Amine Receptors

**Figure 2:**
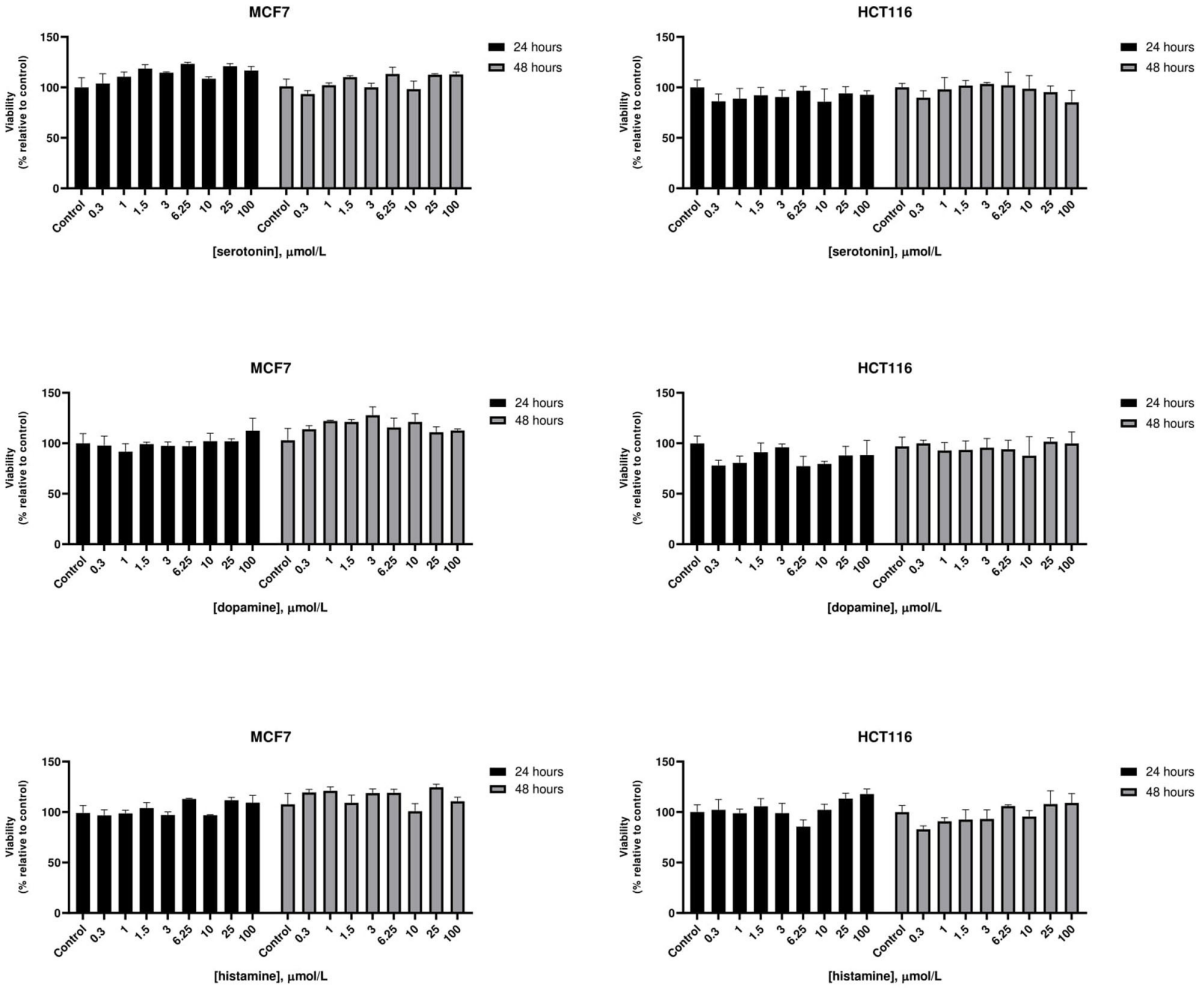
Effect of biogenic amines on HCT116 and MCF7 cell viability. MCF7 and HCT116 cells were grown for 24 or 48 h in serum-free medium in presence of scalar doses of biogenic amines serotonin, dopamine, or histamine. Viabilities were assessed by MTT assay and presented as the percentage of viable cells vs. control. Data show mean ± SD of one representative experiment out of three independent experiments performed in quadruplicate.

At therapeutic concentrations, the main pharmacological targets of these compounds are biogenic amines receptors (37, 38). The precise role of biogenic amines such as histamine, dopamine, and serotonin in cancer is still debated (39–41). To test biogenic amines in our cell lines modes, we treated HCT116 and MCF7 cells with a wide range of concentrations of serotonin, dopamine and histamine and evaluated viabilities after 24 and 48 h. In our assay conditions we observed no significant effect on cell proliferation even at very high doses (Figure 2). Long term treatment of MCF7 cells with the strongest cytotoxic compounds penfluridol, ebastine, pimozide or fluoxetine at clinically significant concentrations determined only a modest increase of drugs efficacy, with IC_50_ values that remained above 3 μmol/L even after 6 days of treatment (Supplementary Figure 3).

**Figure 3:**
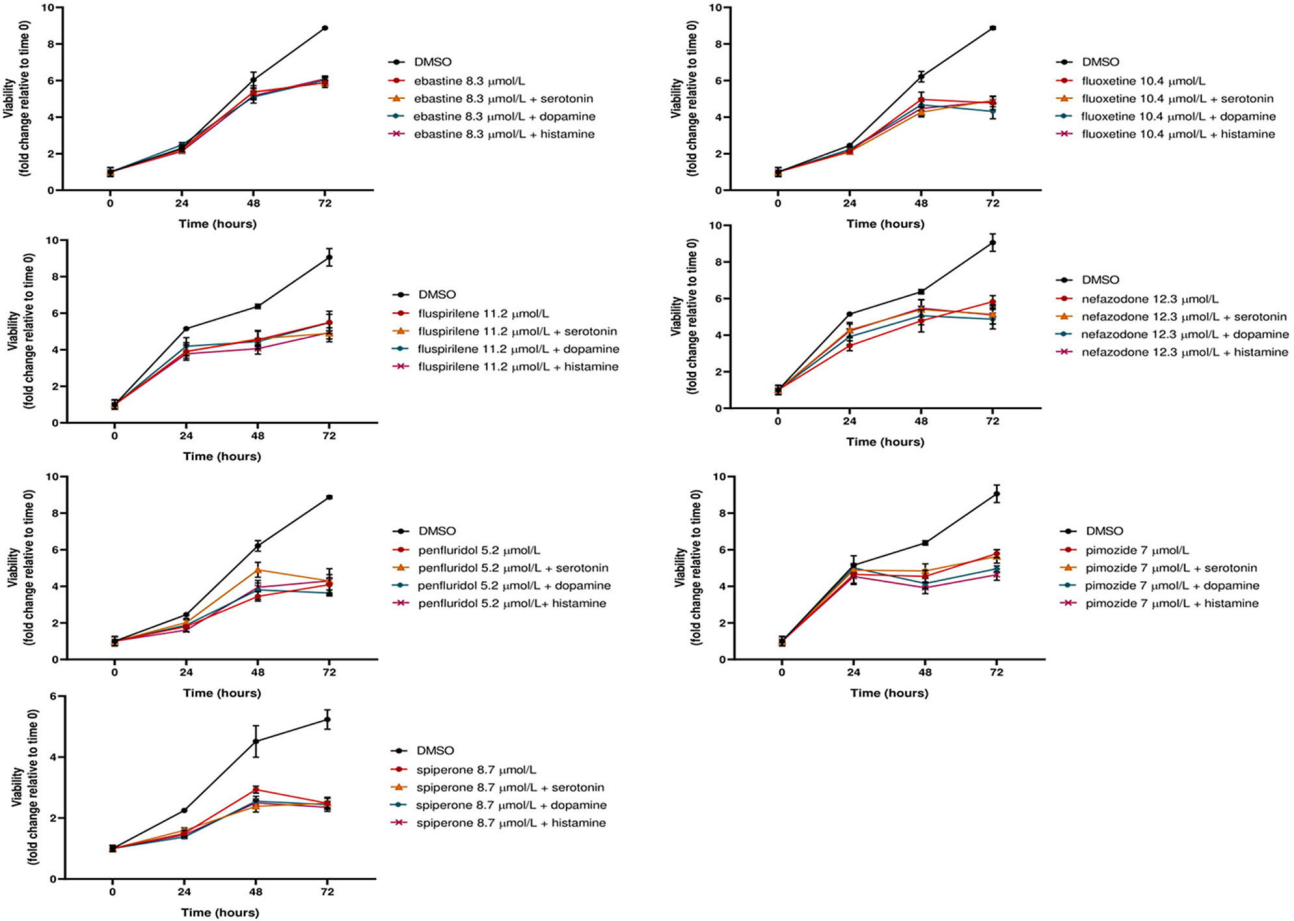
The cytotoxic effect of psychotropic drugs is not reduced by co-treatment with biogenic amines. MCF7 cells were treated with psychotropic drugs alone (at a concentration equivalent to IC_50_) or in presence of 5 μmol/L biogenic amines serotonin, dopamine or histamine. Viabilities were assessed by MTT assay at different time points and presented as fold change relative to control cells treated with vehicle only. Data show mean ± SD of one representative experiment out of three independent experiments performed in quadruplicate.

Notably, neither dopamine, nor serotonin and histamine, added to the culture media, were able to rescue the cytotoxic effect of these drugs (Figure 3).

These data further support the hypothesis that these compounds affect tumor cell viability through a mechanism that is not mediated by the major neuroreceptor systems implicated in their psychotropic effects.

### Psychotropic Drugs Affect Tumor Cell Migration

**Figure 4:**
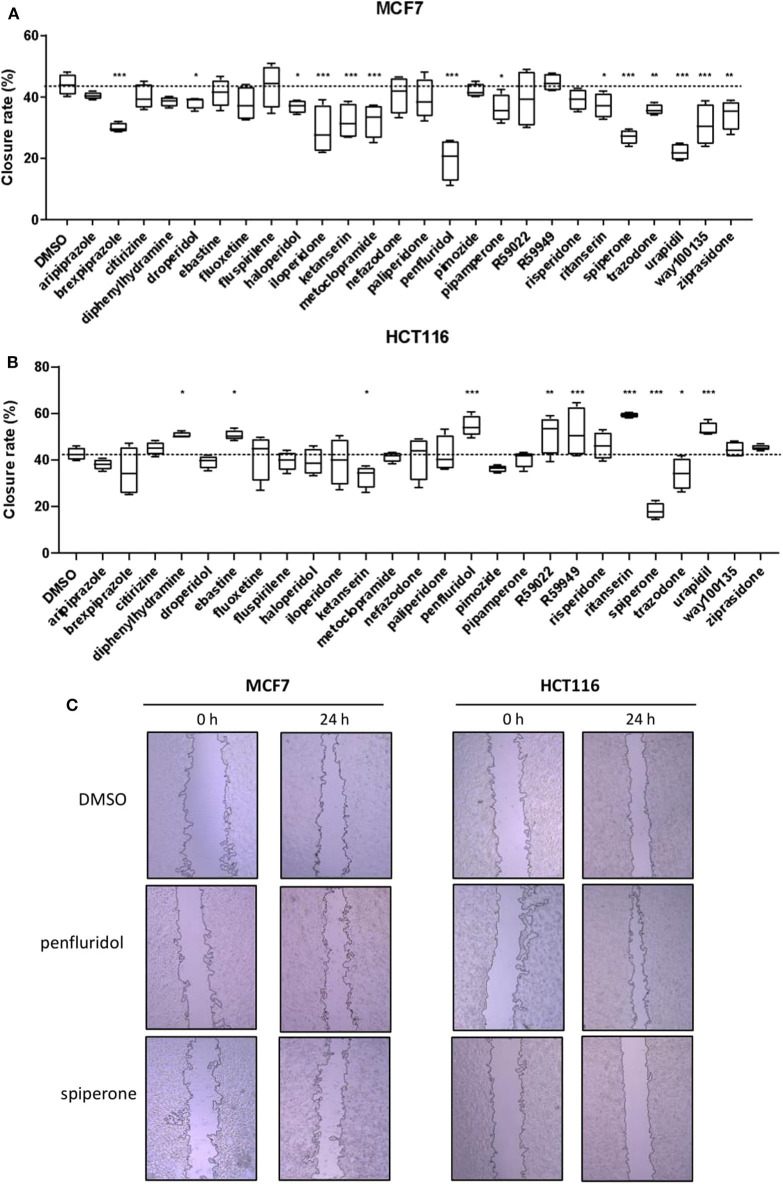
Effect of psychotropic drugs on cancer cells migration. Cell motility was evaluated by wound healing assay. MCF7 **(A)** and HCT116 **(B)** cells were plated in 2 wells IBIDI chambers. After removing the insert, cells were treated with drugs (5 μmol/L) in DMEM 10% FBS. The widths of wounds were measured at 0 and 24 h. Graphs show the closure rate. Data are presented as mean ± SD from three independent experiments, each performed in triplicate. *, Student's *T*-test *p* < 0.05; **, Student's *T*-test *p* < 0.01; ***, Student's *T*-test *p* < 0.001. Representative images of MCF7 and HCT116 wounds after treatment with penfluridol, spiperone, and DMSO **(C)**.

To determine the effect of psychotropic drugs on the motility of cancer cells, we assessed MCF7 and HCT116 cells migration by the wound-healing assay (Figure 4). All active drugs caused a reduction in the motility of MCF7 cells with the strongest effects observed with penfluridol, spiperone, urapidil and brexpiprazole (Figure 4A). On the contrary, the migration rate of HCT116 cells was unexpectedly increased by the cytotoxic compounds ebastine and penfluridol, as well as by different other compounds such as urapidil, diphenhydramine, ritanserin, R59022 and R59949; spiperone, and to a lesser extent, ketanserin and trazodone, reduced HCT116 cells motility (Figure 4B). Overall, these results show that: (i) the impact of the different compounds on the migration rate is not strictly associated with their cytotoxic effect or their conventional pharmacological properties and clinical use; (ii) the effect of the compounds on cell motility is cell line specific.

### Psychotropic Drugs With Significant Antitumoral Activity Display a Cationic Amphiphilic Structure

Cationic amphiphilic drugs (CADs) are defined as chemical compounds with the ability to passively diffuse through lipid bilayers stacking in acid organelles such as lysosomes (42). These compounds contain both a hydrophobic and a hydrophilic domain; the hydrophobic domain contains one or more aromatic rings whereas the hydrophilic part contains a functional amine group that can be ionized (43). CADs family comprises a broad spectrum of compound classes, including dozens of approved drugs that are used to treat a wide range of diseases including allergies, heart diseases, and psychiatric disorders (44, 45). Since the antitumoral activity of compounds investigated in this study is not apparently related to their conventional pharmacological properties and clinical use, we investigated the CADs properties of psychotropic drugs used in our screening evaluating their chemical structure, logP and pKa in comparison to the well-known CADs compounds amiodarone, chlorpromazine and chloroquine (Supplementary Table 2) (46, 47). Since there is not a clear CADs classification based on chemical properties, we set LogP and pKa cut off as suggested by Muehlbacher (36). Overall, 14 psychotropic drugs out of 26 were classified as CADs. Five out of seven most cytotoxic drugs in MCF7 (IC_50_ < 15 μmol/L) were CADs, whereas spiperone and nefazodone, were excluded from CAD classification just because of a LogP or pKa value below the selected cut off (Supplementary Figure 4, Supplementary Table 2). Since CADs were represented also among drugs without cytotoxic activity (e.g., haloperidol, iloperidone, or ritanserin), cationic amphiphilic characteristics contribute strongly, but are not sufficient to confer significant antitumoral activity to psychotropic compounds.

### Psychotropic Drugs Cause Mitochondrial Membrane Depolarization

**Figure 5:**
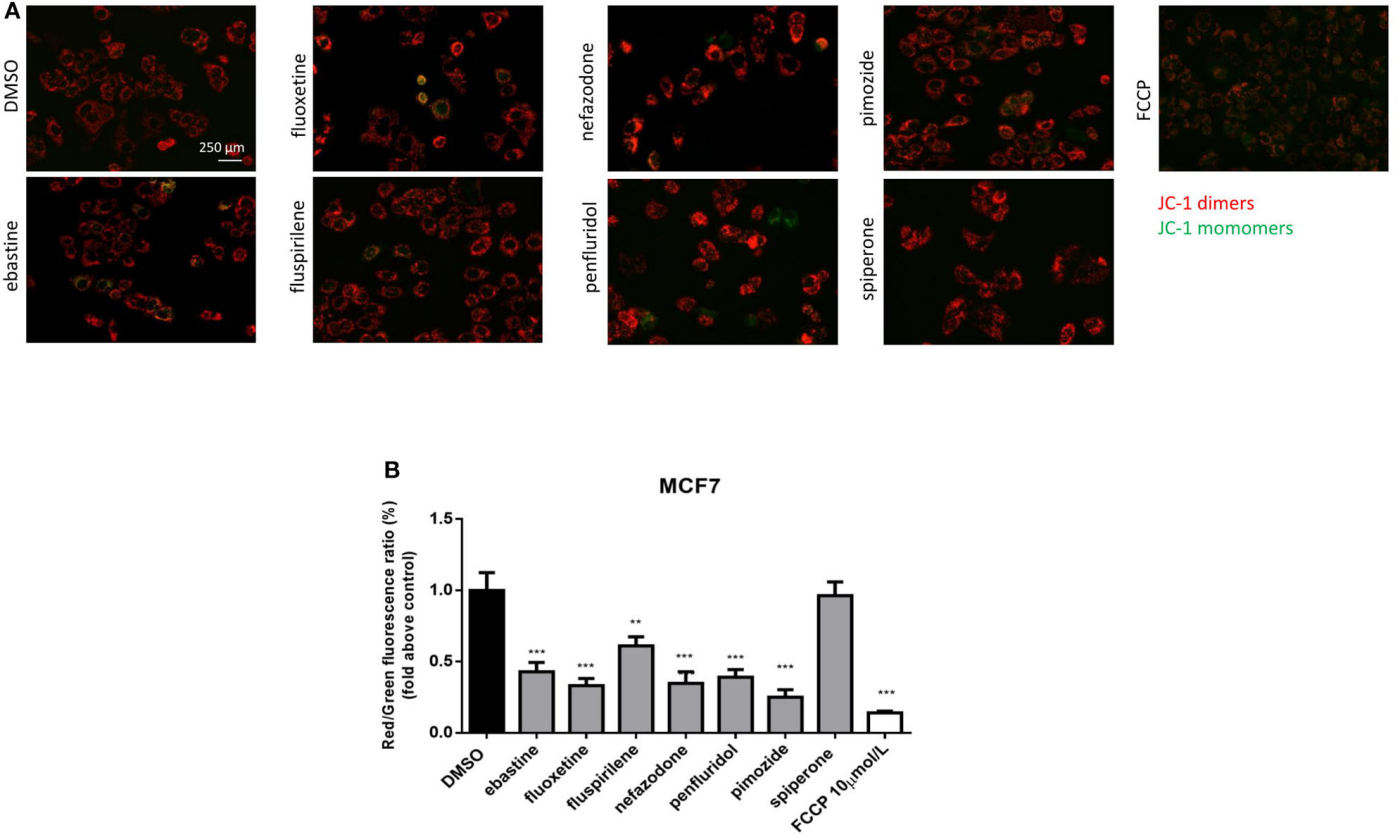
Psychotropic drugs induce mitochondrial membrane depolarization. Mitochondrial membrane potential depolarization was evaluated by JC-1 staining after overnight treatment with psychotropic compounds (5 μmol/L) in MCF7 cells. Pictures were acquired by fluorescence microscopy. Representative images of cell treated with the negative control DMSO, carbonyl cyanide 4-[trifluoromethoxy]phenylhydrazone (FCCP), positive control, ebastine, fluoxetine, fluspirilene, nefazodone, penfluridol, pimozide, and spiperone **(A)**. Histogram showing quantification of red/green fluorescent ratio as fold change relative to control **(B)**. Data are presented as mean ± SD from three independent experiments, each performed in triplicate. **, Student's *T*-test *p* < 0.01; ***, Student's *T*-test *p* < 0.001.

CADs can readily pass through phospholipids bilayers, particularly through membranes with a large transmembrane potential such as the mitochondrial inner membrane. They readily accumulate in the mitochondrial matrix, causing mitochondrial membrane depolarization (45, 48, 49). Therefore, we evaluated the alteration in mitochondrial membrane potential (Δψm) as a function of drug treatment, using the lipophilic cationic dye JC-1 (50). MCF7 cells were treated, for 16 h, with 5 μmol/L of each drug or with FCCP, used as positive control. A significant reduction in Δψm was observed after treatment with ebastine, fluoxetine, penfluridol, pimozide, nefazodone and fluspirilene, but not with spiperone (Figure 5).

### Psychotropics Drugs Induce Vacuolization and Increase Acidic Compartments

**Figure 6:**
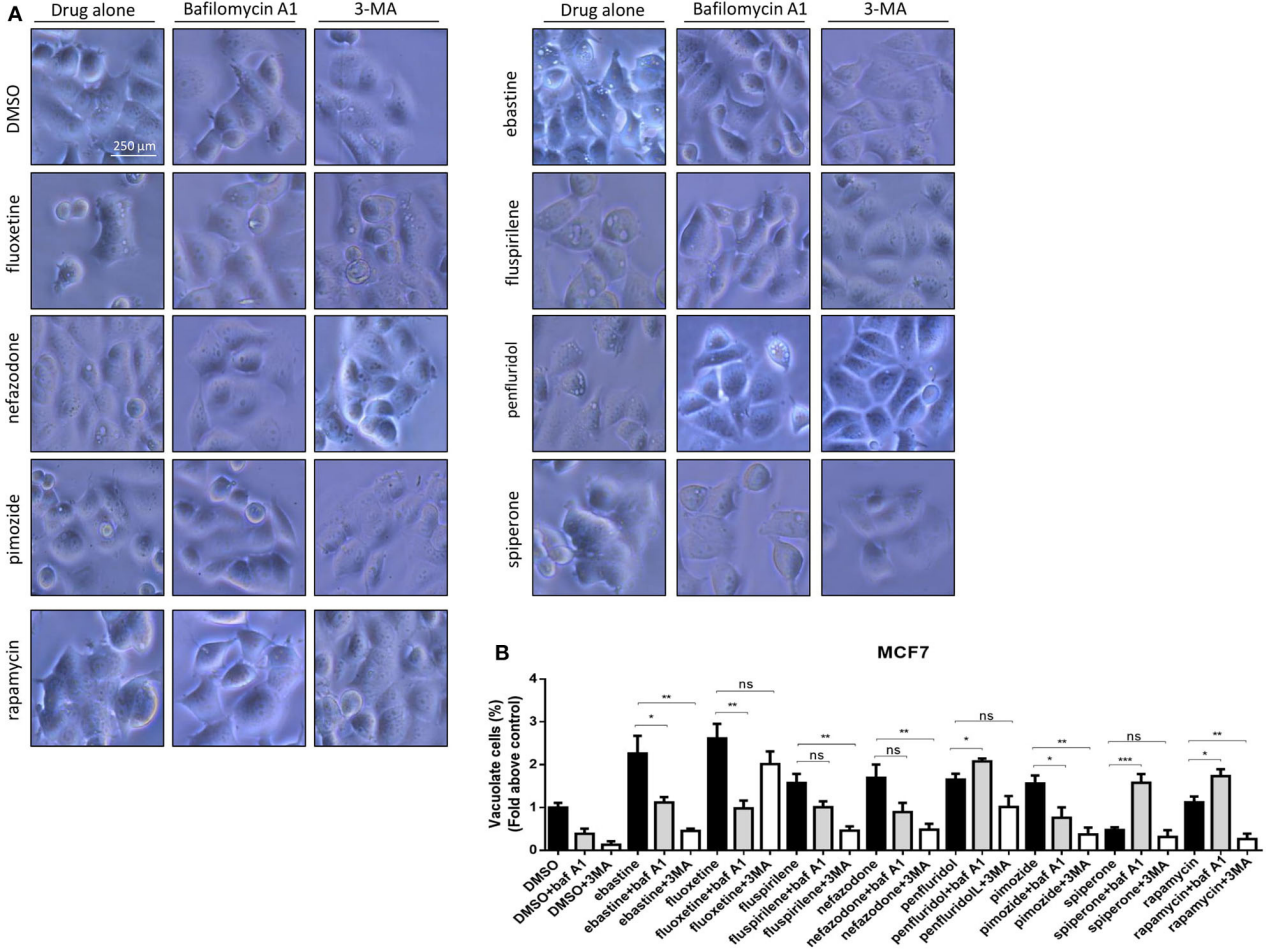
Vacuolar structures formation after treatment of MCF7 cells with psychotropic drugs. Morphological alterations associated with psychotropic drugs treatment in MCF7 were investigated after 4 h exposure by phase contrast microscopy. Representative images of cells treated with the negative control DMSO, ebastine, fluoxetine, fluspirilene, nefazodone, penfluridol, pimozide and spiperone and rapamycin alone or with bafilomycin A1 and 3-MA **(A)**. Histogram showing quantification of vacuoles as fold change relative to control **(B)**. Data are expressed as the mean ± SD of a representative experiment out of three independent experiments performed in triplicate. *p < 0.05; **, Student's *T*-test *p* < 0.01; ***, Student's *T*-test *p* < 0.001.

CADs are known to concentrate in acidic cell compartments because the retro-diffusion of the protonated form is inefficient (mechanism known as ion-trapping or pH partitioning). If sufficiently intense, this sequestration results in the osmotic formation of numerous large, fluid-filled vacuoles already after short term exposure to drugs (46). These molecules are collectively referred to as lysosomotropic agents, for their propensity to concentrate into lysosomes (51). To test the hypothesis that cytotoxic psychotropic drugs concentrate in MCF7 cells by this mechanism, MCF7 were cultured in the presence of 10% FBS and treated with drugs alone or in the presence of the V-ATPase inhibitor bafilomycin A1 or class III PI3K inhibitor 3-MA (Figure 6, Supplementary Figure 5). Fluoxetine induced a strong vacuolar morphology already 6 h after treatment as previously reported (46) (Supplementary Figure 5A); a less prominent, but still significant increase of vacuolar structures was also observed after treatment with fluspirilene, ebastine, pimozide, penfluridol and nefazodone, whereas increase of vacuoles was not observed with spiperone (Supplementary Figure 5). The mTOR inhibitor rapamycin used as a positive control of autophagy induced a mild vacuolar morphology.

In the presence of bafilomycin A1, a significant reduction of vacuoles formation was observed with fluoxetine, ebastine, fluspirilene, pimozide, and nefazodone, suggesting that these drugs require an acidic environment to accumulate and induce vesicles formation; on the contrary, a higher number of vesicles was observed after treatment with penfluridol and spiperone, suggesting that these drugs do not require pre-existing acidic compartments to induce vacuolization although they can cause the formation of autophagosome structures that accumulate after inhibition of autophagosome-lysosome fusion and autolysosome acidification by bafilomycin A1 (Figure 6). The autophagosome nature of vacuoles induced by all these compounds was supported by the reduction of the number of vesicles in the presence of the class III PI3K inhibitor 3-MA (Figure 6).

**Figure 7:**
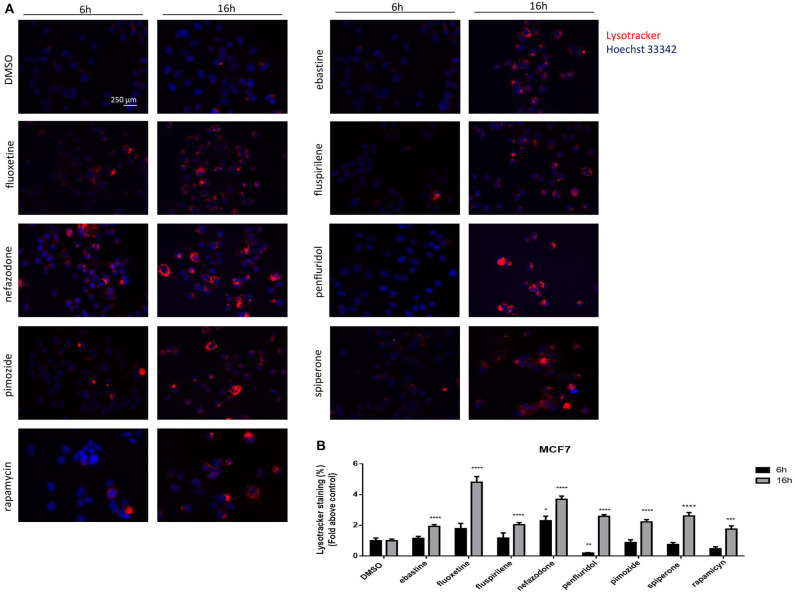
Psychotropic drugs induce acidic compartment formation perturbing lysosomal and autophagic functioning. Effects of psychotropic drugs on intracellular acidic compartments were evaluated by Lysotracker Deep Red staining after 6 and 16 h of treatment. Nuclei were stained using Hoechst 33342. Pictures were acquired by fluorescence microscopy (magnification: 20×). Representative images of cells treated with DMSO, negative control, rapamycin, positive control, ebastine, fluoxetine, fluspirilene, nefazodone, penfluridol, pimozide, and spiperone **(A)**. Graphs showing quantification of red lysotracker staining/blue nuclei staining ratio as fold change relative to negative control **(B)**. Data are expressed as the mean ± SD of a representative experiment out of three independent experiments performed in triplicate. *p < 0.05; **, Student's *T*-test *p* < 0.01; ***, Student's *T*-test *p* < 0.001; ****p < 0.0001.

The nature of the vacuoles induced by psychotropic drugs was further investigated by staining MCF7 cells with the LysoTracker dye, which is a highly soluble small molecule that is retained in acidic subcellular compartments, such as late endosomes and lysosomes, whose presence is an indirect indication for autophagic activity (52). Although a transient increase of pH in autophagosome-lysosome structures was observed after short term treatment with penfluridol (Figure 6B), LysoTracker dye staining clearly shows a strong increase of acidic compartments after overnight treatment with all drugs tested, consistent with increased autophagosome-lysosome acidic structures (Figure 7, Supplementary Figure 6).

### Spiperone and Penfluridol Induce Autophagy by Modulating mTOR and AMPK Pathways

**Figure 8:**
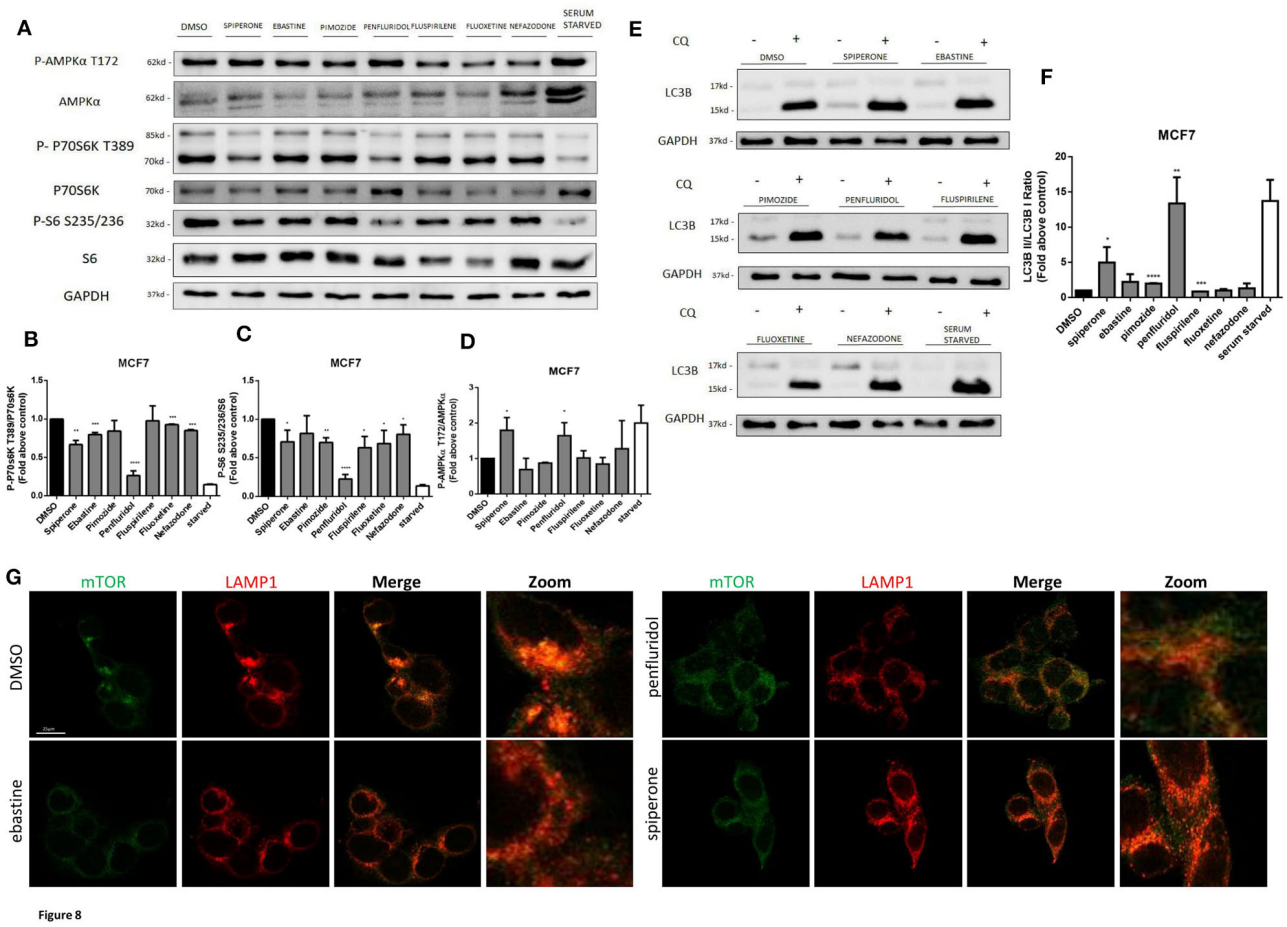
Effect of psychotropic drugs on mTOR and AMPK pathways and autophagic flux. Western blot analysis of MCF7 cells after 16 h treatment with psychotropic drugs. Lysates were analyzed for p-AMPKα T172, AMPKα, p-P70S6K T389, P70S6K, P-S6 S235/236, S6, LC3B, and GAPDH **(A)**. Histogram showing quantification of P70S6K **(B)**, S6 **(C)** and AMPK **(D)** phosphorylation normalized on total protein P70S6K, S6 and AMPKα, respectively. WB **(E)** and histogram **(F)** showing the relative expression of LC3B II/I upon chloroquine treatment. Densitometric analyses are expressed as the mean ± SD of three independent experiments performed in triplicate. * Student's *T*-test *p* < 0.05 **, Student's *T*-test *p* < 0.01; ***, Student's *T*-test *p* < 0.001, ****p < 0.0001. Delocalization of mTOR from the lysosomal membrane was evaluated in MCF7 after 16 h treatment with psychotropic drugs. mTOR was stained using mTOR primary antibody and Alexa Fluor 488 secondary antibody (green). Lysosomes were stained using LAMP1 primary antibody and Alexa Fluor 536 secondary antibody (red). Representative images of DMSO, negative control, ebastine, penfluridol, and spiperone **(G)**.

The increase of acidic structures can be a consequence of both autophagy induction and reduced turnover in the autophagosomal compartment caused by impaired autophagosome-lysosome fusion and/or lysosomal function. In order to clarify this issue, we investigated the main regulators of autophagy: mTOR pathway (represented by phosphorylations in 70S6K T389 and ribosomal protein S6 S235/236) and AMPK activation (Figure 8). Starvation, a strong inducer of autophagy, was used as positive control. A strong inhibition of mTOR pathway, comparable to that obtained with starvation, was observed after treatment with penfluridol, whereas a milder but significant downregulation of the pathway was detected with spiperone and, to a lesser extent, with the other compounds (Figures 8A–C), since S6 Ser 235/236 phosphorylation might also be modulated by kinases different from P70S6K (53). Notably, a partial delocalization of mTOR from the lysosomal membrane, further supporting mTOR inhibition, was observed after treatment with both penfluridol and spiperone (Figure 8G, Supplementary Figure 7).

In agreement with mTOR dislocation, a significant increase of AMPK phosphorylation in the activation site T172, comparable to that induced by starvation, was observed after treatment with penfluridol and spiperone. On the contrary, AMPK phosphorylation was unaffected or slightly reduced after treatment with all other compounds (Figures 8A,D).

The conversion of the cytosolic LC3B form, LC3B-I, into the faster migrating, phosphatidylethanolamine-conjugated, LC3B-II form, a marker of autophagy induction (54) was significantly enhanced in cells treated with penfluridol, spiperone and pimozide (Figures 8E,F).

### Psychotropic Drugs With Cationic Amphiphilic Properties Cause Lysosomal Disruption

**Figure 9:**
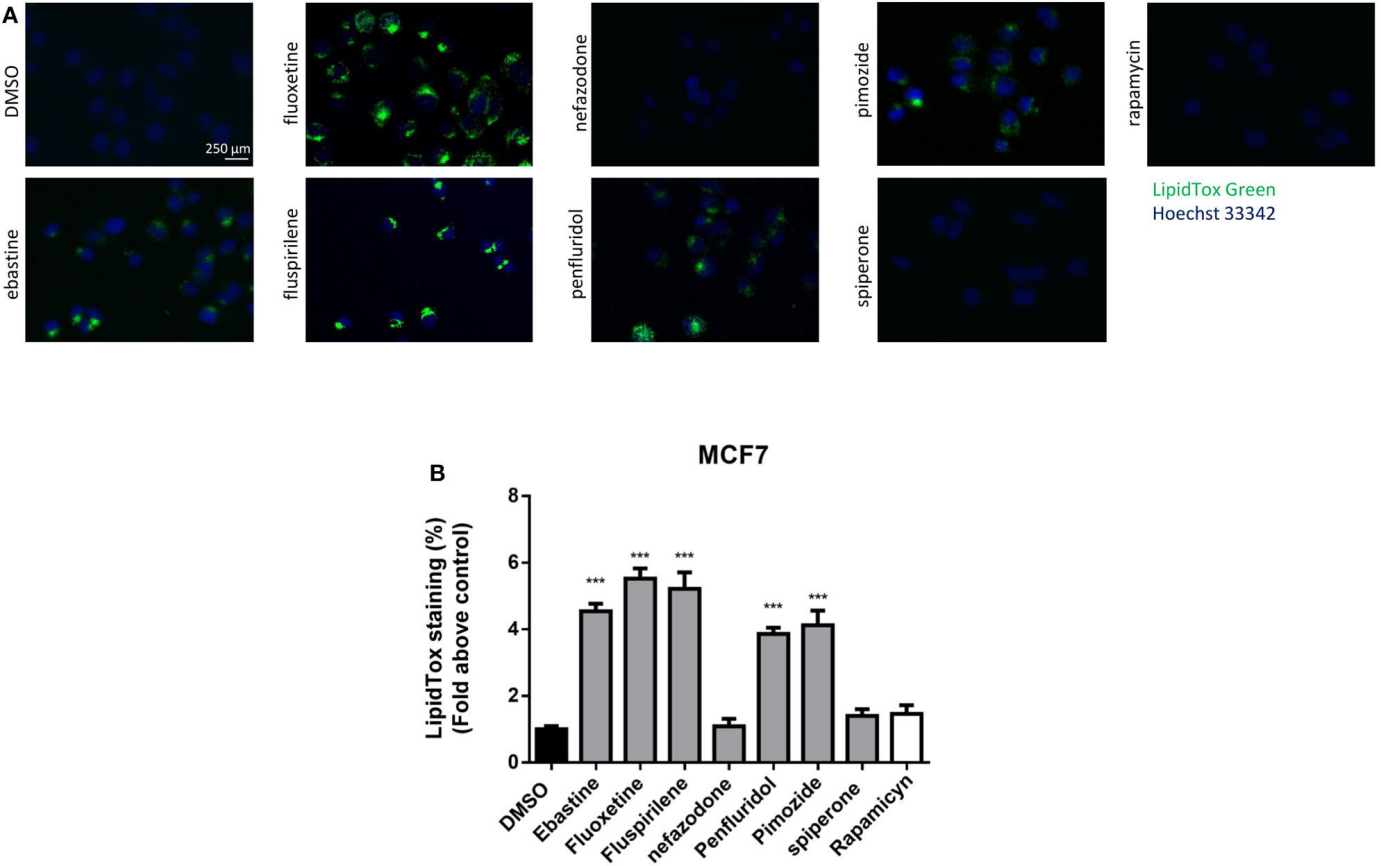
Treatment with psychotropic drugs induces phospholipidosis in MCF7 cells. Accumulation of phospholipids in MCF7 cell line was evaluated after 16 h treatment with drugs using LipidTox green staining. Nuclei were stained using Hoechst 33342. Pictures were acquired by fluorescence microscopy (magnification: 20×). Representative images of cells treated with DMSO, negative control, ebastine, fluoxetine, fluspirilene, nefazodone, penfluridol, pimozide, spiperone, and rapamycin **(A)**. Histogram showing quantification of Green LipidTox staining/blue nuclei staining ratio as fold change relative to control **(B)**. Data are presented as mean ± standard deviation from three independent experiments, each performed in triplicate. ***, Student's *T*-test *p* < 0.001.

CADs can accumulate into lysosomes and impair lysosomal enzymatic activities (44, 55). It has also been shown that several antipsychotic and antidepressant drugs extensively accumulate in lysosomes and inhibit acid sphingomyelinase and phospholipases (36, 56). Lysosomes are a major site of cellular membranes degradation and complex lipids metabolism, therefore the hallmark of drug-induced lysosomal impairment is accumulation of phospholipids (42, 57). Therefore, we investigated whether the antitumoral activity of psychotropic drugs was associated with lysosomal impairment by incubating cells in the presence of phospholipids conjugated to fluorescent dye. After incubation for 24 h with LipidTOX, MCF7 cells treated with ebastine, fluspirilene, fluoxetine, pimozide and penfluridol showed a strong increase of phospholipids aggregates; on the contrary, this phenotype was not observed after treatment with non-CADs spiperone and nefazodone and with the inducer of autophagy rapamycin (Figures 9A,B).

**Figure 10:**
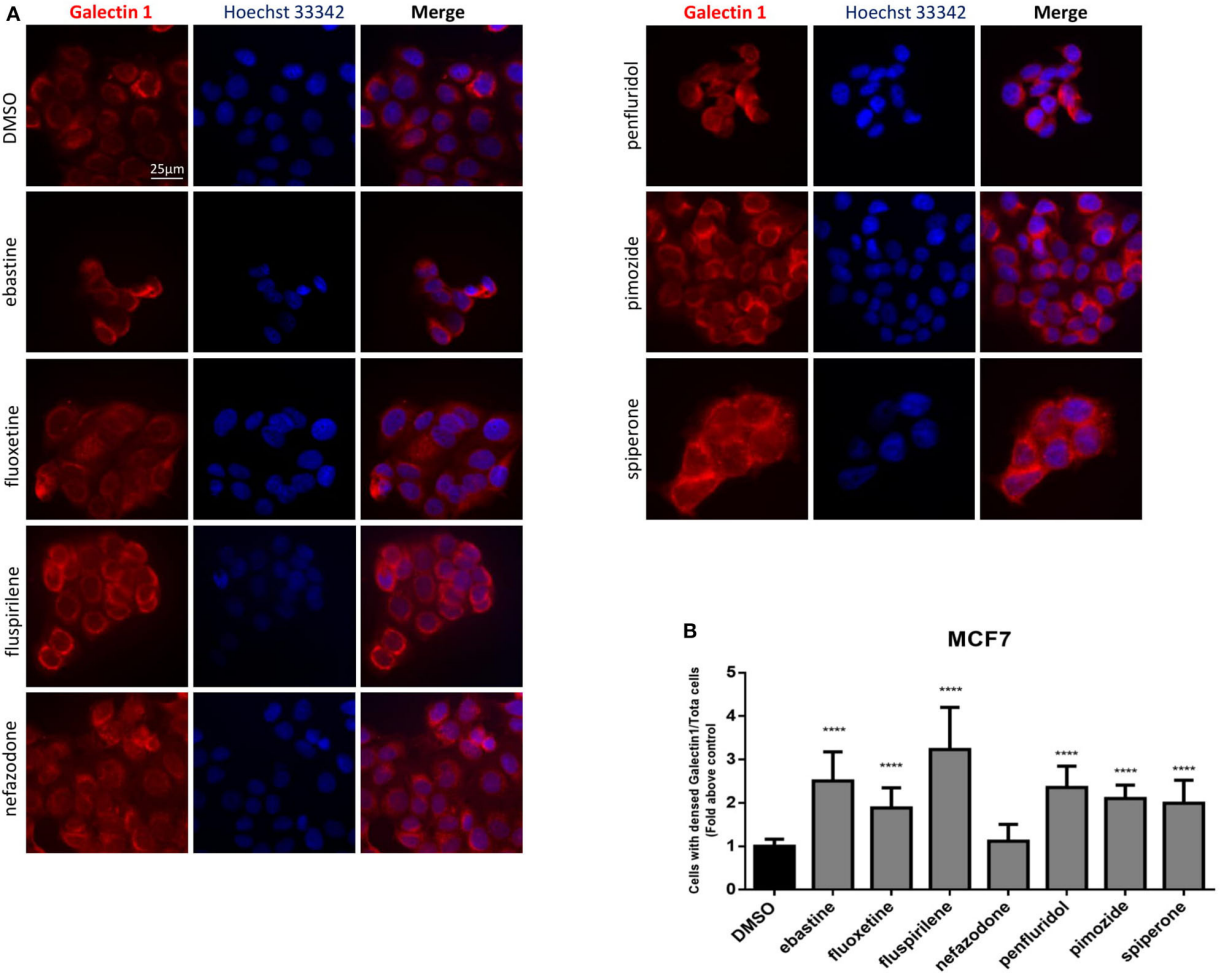
Treatment with psychotropic drugs induced formation of galectin-1 complexes. Formation of galectin-1 complexes in MCF7 after 16 h treatment with psychotropic drugs was observed by fluorescence microscopy (magnification: 63X). Galectin-1 was stained with anti galectin-1 primary antibody and Alexa Fluor 563 secondary antibody. Nuclei were stained using DAPI. Representative images showing cells treated with psychotropic drugs: DMSO, ebastine, fluoxetine, fluspirilene, nefazodone, penfluridol, pimozide, and spiperone **(A)**. Histogram showing the number of cells presenting galectin-1 complexes/total number of cells ratio as fold change relative to control **(B)** Data are presented as mean ± standard deviation from three independent experiments, each performed in triplicate. ****p < 0.0001.

Drugs with cationic amphiphilic properties accumulating into lysosomes can also induce LMP. This phenomenon can lead to the release of lysosomal enzymes inside the cytoplasm and possibly cell death (17). Galectin-1 is a small protein normally located in the cytoplasm and in the nucleus, that accumulates and forms complexes to the lysosomal membrane in case of lysosomal membrane damage and LMP (58). To evaluate lysosomal membrane damage in response to psychotropic drug treatment we investigated galectin-1 complex formation by immunofluorescence. The formation of galectin-1 complexes was observed with all the drugs tested, apart from nefazodone, indicating that cytotoxic psychotropic drugs can induce lysosomal membrane damage (Figures 10A,B).

### Psychotropic Drugs Induce Different Types of Cell Death

**Figure 11:**
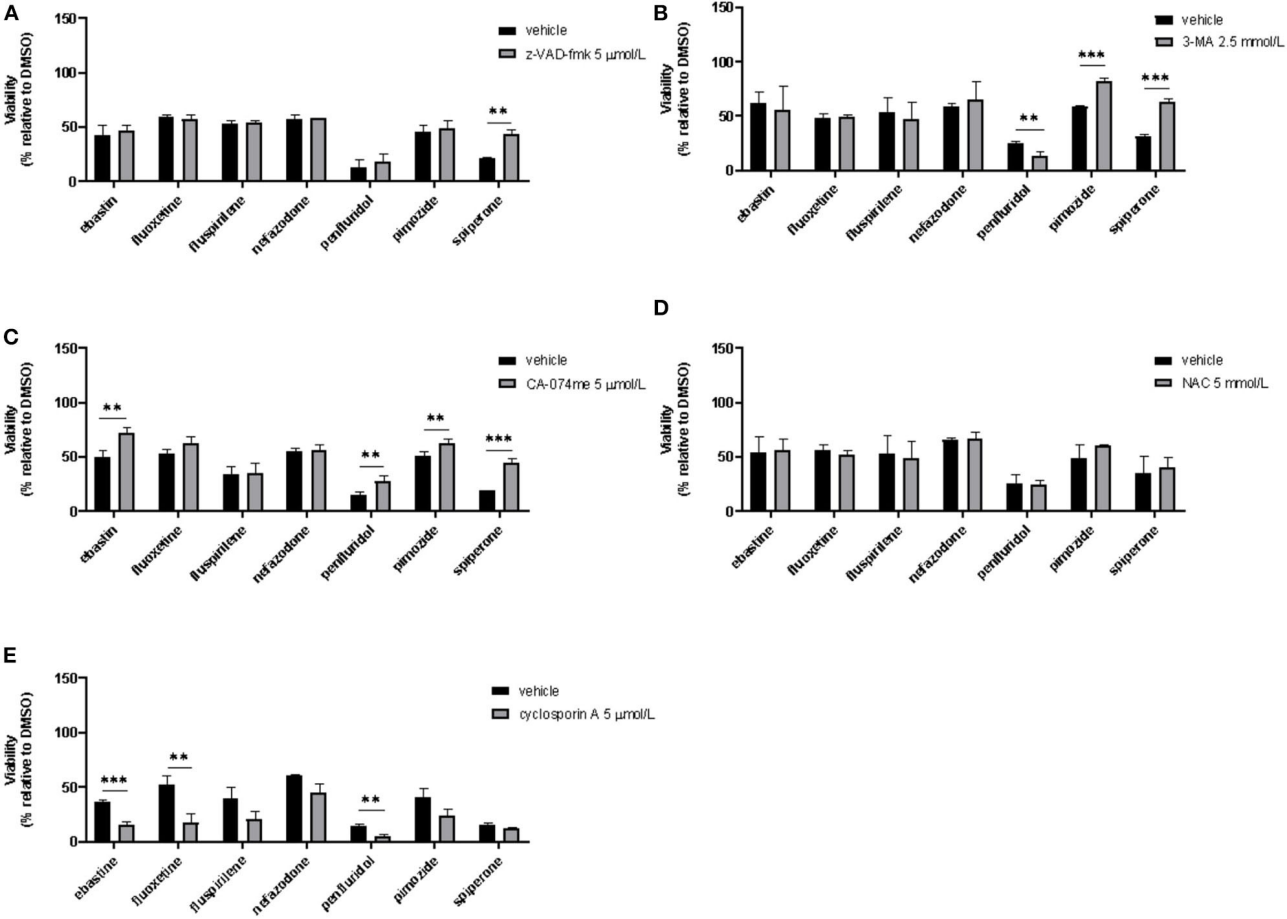
Effect of co-treatment of psychotropic drugs and the pan-caspase inhibitor zVAD-fmk, the autophagy inhibitor 3-MA, the cathepsin inhibitor CA-074 me, the antioxidant NAC or the inhibitor of mitochondrial membrane depolarization cyclosporin A. MCF7 cells were treated for 72 h with vehicle or psychotropic drugs (all 10 μmol/L except penfluridol, 5 μmol/L) alone, or in combination with zVAD-fmk, 5 μmol/L **(A)**, 3-MA, 2.5 mmol/L **(B)**, CA-075 me, 5 μmol/L **(C)**, NAC, 5 mmol/L **(D)**, cyclosporin A, 5 μmol/L **(E)** Data show mean ± SD of at least three independent experiments performed in triplicate. The graphs show cell viability as the percentage of viable cells vs. control. **, Student's *T*-test *p* < 0.01; ***, Student's T-test *p* < 0.001.

To assess if apoptosis is involved in psychotropic drugs-induced cell death we performed PI/Annexin V staining in MCF7 cells. FACS analysis at different time points showed an increase in necrosis cells with all the drugs but a significant induction of apoptosis after 48 h of treatment with the sole spiperone (Supplementary Figure 8). These data were further confirmed by viability rescue experiments with a pan caspase inhibitor zVAD-fmk. As shown in Figure 11A, zVAD-fmk significantly rescued cell death only in cells treated with spiperone and staurosporine, whereas it was ineffective with the other drugs.

Since apoptosis is not the primary mechanism of death elicited by cytotoxic psychotropic drugs, except for spiperone, we investigated the role of autophagy by treating cells with the autophagy inhibitor 3-MA (59). As shown in Figure 11B, 3-MA co-treatment significantly rescued cell viability in cells treated with rapamycin and in cells treated with spiperone and pimozide. Conversely, 3-MA enhanced penfluridol cytotoxicity, whereas it did not show any effect in combination with ebastine, fluoxetine, nefazodone and fluspirilene. However, since it was reported that in particular conditions 3-MA could induce autophagy (59) we performed western blot analysis to investigate the conversion of the cytosolic LC3 I to II form in MCF7 cells treated with spiperone and penfluridol alone or in combination with 3-MA (Supplementary Figure 9). Our data indicate that in our experimental set-up 3-MA does not induce autophagy, on the contrary it is effective in suppressing LC3 II conversion.

To further investigate the mechanism of the observed cytotoxicity we assessed whether inhibition of lysosomal cathepsins B and L rescued cell viability in MCF7 cells, for this purpose we performed experiments with the inhibitor CA-074 me (60). As displayed in Figure 11C CA-074 me significantly rescued cell death induced by ebastine, penfluridol, pimozide and spiperone, while a mild but not significant effect was observed in cells co-treated with fluoxetine.

Additionally, in order to clarify if oxidative stress was involved in psychotropic drugs-induced cell death, we co-treated MCF7 cells with the antioxidant NAC, however no significant effect was observed in terms of viability rescue (Figure 11D). With cyclosporin A, an inhibitor of the mitochondrial permeability transition pore (mPTP), an additive cytotoxic effect was observed with all drugs tested (Figure 11E). Cyclosporin A has been reported to be a broad-spectrum multidrug resistance modulator (61) and this activity possibly induces psychotropic drugs retention resulting in a boost of cytotoxicity.

## Discussion

Although cancer treatment has witnessed remarkable progress over the past few decades, cancer remains a major threat to humans, with total cure remaining elusive. Repurposing of well-characterized and well-tolerated drugs for cancer therapy has emerged as an attractive alternative for a long and costly process of drug development (23). Psychotropic drugs are revealing promising candidates for drug repositioning in cancer. Although several *in vitro* and *in vivo* models reported the efficacy of this family of drugs in reducing cancer cell viability and tumor growth (30, 32, 62), the pharmacological properties underpinning the possible clinical application of psychotropic drugs for cancer therapy remain poorly understood. In this study we investigated a large panel of psychotropic drugs for their potential anti-tumoral activity evaluating their cytotoxic effect in six cell lines derived from three different tumor types. By using stringent screening conditions, we identified only a few compounds that significantly reduced cell viability at clinically relevant concentrations. These were represented by the antipsychotics penfluridol, pimozide, fluspirilene, nefazodone, and spiperone, the antidepressant fluoxetine and the antihistamine ebastine. Except for spiperone, whose cytotoxicity was negligible in GB, all the other compounds showed cytotoxic activity in all cell lines tested.

The comparable efficacy, in three different tumor types, of compounds with clinically different indications allows us to speculate a common mechanism of action independent from the phenotypic and molecular profile of the tumor and not associated with the conventional pharmacological properties and clinical use of these compounds. This hypothesis is corroborated by the negligible cytotoxicity observed with other drugs with superimposable biogenic amine receptors targeting, by the lack of rescue of cell viability after co-treatment with biogenic amines and by the drug concentration necessary to observe a biologic effect, that it is at least one order of magnitude higher than that needed for their conventional pharmacological targets (63).

Based on the analysis of structure and chemical-physical properties, most psychotropic compounds with a significant cytotoxic activity can be classified as CADs (36, 43). It is well-demonstrated the formation of cytoplasmic vesicles in cells exposed to CADs results from extensive ion-trapping-based accumulation of lysosomotropic weak bases in acidic compartments (36, 55). Vacuoles formation, inhibited by the disruption of the lysosomal V-ATPase, was observed after short term exposure of MCF7 cells to CADs fluoxetine, ebastine, fluspirilene, pimozide but also to nefazodone, that is not formally a CADs but might display some of their features. Accumulation of vacuoles in the presence of bafilomycin A1 was instead observed after treatment with penfluridol and spiperone, suggesting that the formation of vesicles by these drugs does not necessarily depend on ion-trapping in acidic compartments, but is favored by the block of lysosomal activity. The acidic autophagosome nature of these vesicles was confirmed by the requirement of class III PI3K for their formation and by the positive staining with the lysosomotropic dye LysoTracker. Notably, both spiperone and penfluridol, that induced the formation of autophagosome structures independently from the ion-trapping mechanism are likely true activator of autophagy, as demonstrated by stimulation of AMPK and LC3B conversion and downregulation of mTOR pathway observed in MCF7 cells.

Although lysosomotropic CADs can increase lysosomal pH after compound sequestration which could lead to suboptimal conditions for lysosomal digestion (64, 65), lysosomal pH increase may be a transient change and pH could be restored after extended exposure to lysosomotropic compounds (47, 66, 67). The increased LysoTracker dye staining we observed after overnight treatment with drugs indicates a pH recovery after compound sequestration and reflects the increased lysosomal volume, suggestive of the occurrence of lysosome biogenesis induced by lysosomotropic drugs (47, 68). Moreover, drug interactions with the lysosomal lipid bilayer and membrane proteins could influence the dynamics of membrane fusion and/or fission, thereby affecting trafficking steps and lysosomal egress (67), causing a reduction in autophagic flux and lysosomal enlargement.

Due to their chemical structure, CADs can accumulate in acidic lysosomes (46) and incorporate to luminal membranes where they function as effective inhibitors of acid sphingomyelinase and other lysosomal lipases (36, 44). At therapeutically relevant concentrations, CADs have been shown to cause the lysosomal accumulation of various lipid species, including sphingomyelin, phosphatidylethanolamine, phosphatidylserine, phosphatidylcholine, lysophosphatidic acid, and cholesterol, with induction of phospholipidosis (42, 57). In our experimental model, CADs ebastine, fluspirilene, fluoxetine and pimozide, that very rapidly accumulated in cells by ion-trapping, caused a strong increase of phospholipids aggregates. Our observations are supported by papers reporting the capacity of these compounds to induce phospholipidosis. Gonzalez-Rothi in 1995 first described the complication of pulmonary phospholipidosis in a patient with manic-depressive illness after treatment with fluoxetine (69); penfluridol, pimozide, and fluspirilene have been reported in a screening of drugs capable to inhibit sphingomyelinase and were found to induce phospholipidosis in neuroglioma H4 cells (36, 44), whereas ebastine was identified by electron microscope screening to evaluate chemicals for drug-induced phospholipidosis (70). Our results demonstrate that, also in cancer cells, ebastine, fluspirilene, fluoxetine and pimozide act as typical CADs, impairing lysosomal activity.

Some compounds investigated in this study, including the antipsychotics diphenylbutylpiperidines fluspirilene, penfluridol, and pimozide and antidepressants such as fluoxetine have been previously reported as autophagy inducers in neurons and in different cancer cell types such as BC and GB by affecting a variety of targets (31, 71–73). Our study shows that the cytotoxic activity of most of these compounds is essentially based on their common cationic amphiphilic properties and their capacity to perturb acidic intracellular compartments. Moreover, although all investigated drugs caused the formation of acidic structures, apparently inducing the autophagic flux, only spiperone, penfluridol and, potentially, pimozide can be considered true autophagy activators. Overall, these data raise a critical issue related to clinical use of these compounds as autophagy enhancers, but they also reveal interesting therapeutic implications for compounds that transiently increase upstream autophagic flow while compromising downstream lysosomal function.

The lysosome is emerging as a driving force in the progression of numerous human cancers, in which enhanced function of the autophagy–lysosome system enables efficient nutrient scavenging and growth in nutrient-poor microenvironments, promote the metastatic potential and treatment resistance (11). But lysosomal activation in aggressive cancers can lead to alterations in lysosomal structure and function, which, paradoxically, renders cancer cells more sensitive to lysosomal destabilization (5, 74). This frailty can be targeted by lysosomotropic compound that may have an antitumor effect preferentially killing the more sensitive cancer cells by inducing dysregulation of lysosomal lipid metabolism and LMP with release into the cytosol of cathepsins, potent inducers of cell death (17, 75, 76). In our study, we observed increased Lysotracker staining, suggestive of lysosomal swelling that is considered a typical condition preceding LMP (17, 77–79) and galectin-1 complexes, a surrogate marker of lysosomal membrane damage (58), suggesting a possible role of lysosomes in cancer cell death. This was confirmed for ebastine, penfluridol, pimozide, and fluoxetine, whose cytotoxic activity was partially rescued by inhibitor of cathepsins B and L but not by treatment with both apoptosis or autophagy inhibitors.

Inhibition of apoptosis and autophagy were also ineffective in reducing cell death induced by nefazodone and fluspirilene and further experiments are required to clarify the mechanisms of cell death induced by these drugs.

Notably, while inhibition of autophagy significantly rescued pimozide and spiperone cytotoxicity, it further increased cell death induced by penfluridol, the compound that demonstrated the highest cytotoxicity in all cell lines tested. The strong antitumoral activity of penfluridol may be due to its ability to induce both ADCD and LMP. Most of the known compounds that affect autophagy in neoplastic cells are either inducers or inhibitors of this process (80, 81). However, molecules that can modulate autophagy in a dual mode, by both inducing and inhibiting the process, seem to represent a novel and effective strategy for anticancer therapy (82, 83).

Finally, all psychotropic compounds with cationic amphiphilic properties caused a significant reduction in Δψm. Since oncogenic activation leads to increased mitochondrial metabolism and higher Δψm compared to that of non-cancer cells (20) and experimental evidence demonstrates that irreversible mitochondrial membrane depolarization can induce cell death also in apoptotic resistant cells (84), CADs appear excellent candidates for mitochondrial targeting in cancer, as they can easily diffuse in tumor tissues and interact with negatively charged mitochondrial membranes (20, 45, 49). Since in our cell line model cytotoxicity of psychotropic drugs was not mediated by ROS and thiols oxidation whereas apoptosis has been demonstrated only in cells treated with spiperone, studies are underway to explore the molecular mechanisms underlying CADs induced mitochondrial membrane depolarization and its role in inducing cancer cell death.

In addition to acute cytotoxicity, observed, *in vitro*, at lower micromolar concentrations, *in vivo* psychotropic drugs with cationic amphiphilic properties can also impair cancer cell metabolism and sensitize tumors to chemotherapy at plasma concentrations achieved with standard therapeutic regimens (85, 86). Suggestive of their efficacy in human clinical setting, epidemiologic studies have reported a reduced incidence of glioma and CRC among users of tricyclic antidepressants (27), a lower CRC risk under therapy with fluoxetine (26, 87) and an association between post-diagnostic use of cationic amphiphilic antihistamines and reduced cancer mortality as compared with similar use of antihistamines that do not classify as CADs (88).

In conclusion, the data presented above identify a subset of psychotropic drugs as putative anticancer agents and open a feasible, safe, and economically sound possibility to test the clinical anticancer efficacy of this therapeutic class of compounds. In particular, the cytotoxicity of psychotropic drugs with cationic amphiphilic structures relied on simultaneous mitochondrial and lysosomal disruption and induction of cell death that not necessarily requires apoptosis. Since dual targeting of lysosomes and mitochondria constitutes a new promising therapeutic approach for cancer, particularly those in which the apoptotic machinery is defective, these data further support their clinical development.

## Data Availability Statement

The raw data supporting the conclusions of this article will be made available by the authors, without undue reservation.

## Author Contributions

MV and AA equally contributed in study design, conducting experiments, acquiring data, analyzing data, and writing the manuscript. VB contributed in data analysis and revising the manuscript. KR, VY, and AV contributed in conducting experiments. AM and GB contributed in data interpretation and manuscript editing. DC contributed in study design, data interpretation, manuscript writing, and the final approval of the manuscript. All authors critically reviewed and agreed on the final version of the manuscript.

## Conflict of Interest

The authors declare that the research was conducted in the absence of any commercial or financial relationships that could be construed as a potential conflict of interest.
