# Supplementary material for: Psychotropic Drugs Show Anticancer Activity by Disrupting Mitochondrial and Lysosomal Function
Source: Front Oncol. 2020 Oct 19;10:562196. doi: 10.3389/fonc.2020.562196 (PMC7604408; doi:10.3389/fonc.2020.562196)
Supplement: Supplementary file 1 [file Data_Sheet_1.pdf]

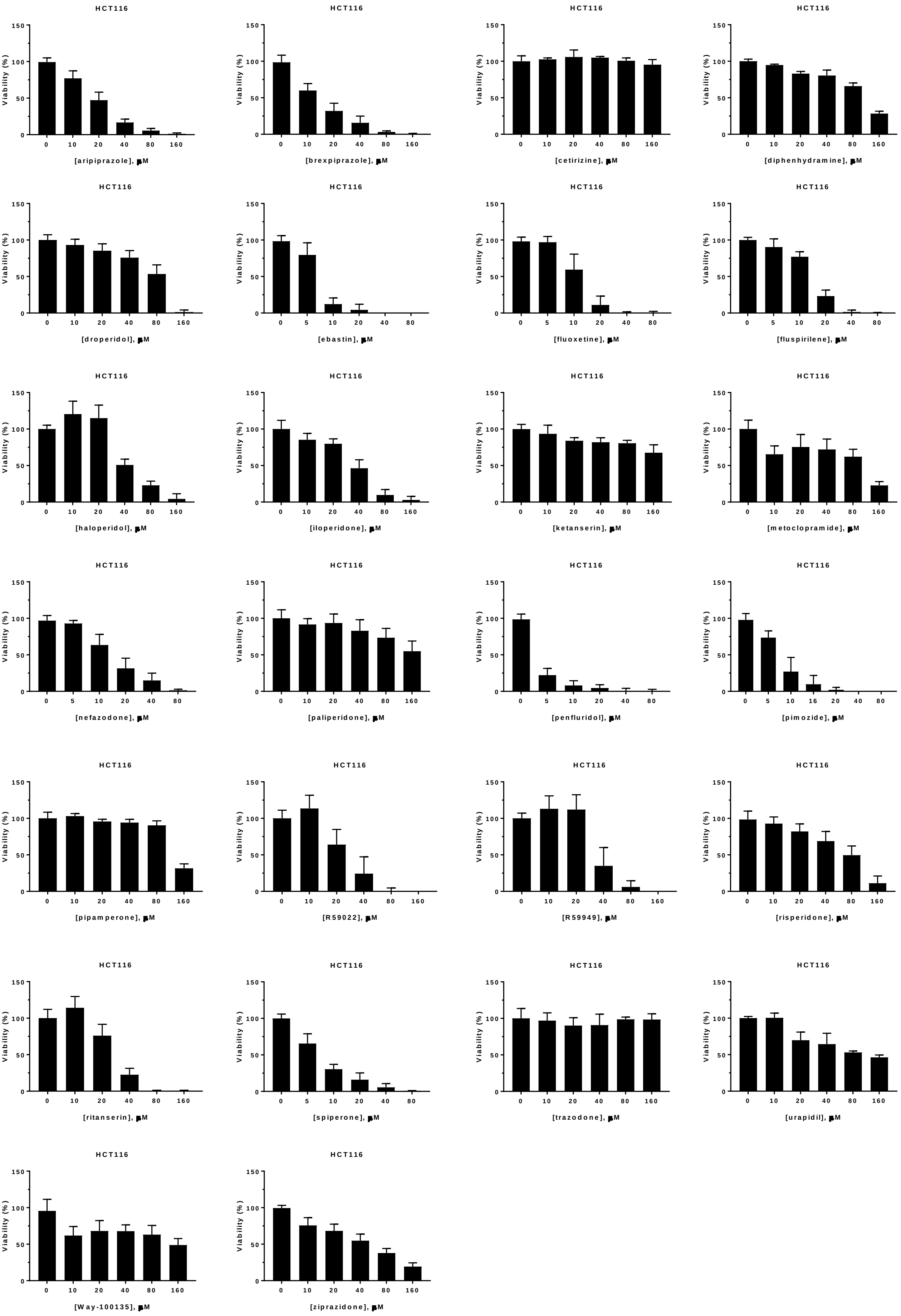
HCT116
Viability (%)
150 100 50 0
0 10 20 40 80 160
[aripiprazole], µM
HCT116
Viability (%)
150 100 50 0
0 10 20 40 80 160
[brexpiprazole], µM
HCT116
Viability (%)
150 100 50 0
0 10 20 40 80 160
[cetirizine], µM
HCT116
Viability (%)
150 100 50 0
0 10 20 40 80 160
[diphenhydramine], µM
HCT116
Viability (%)
150 100 50 0
0 10 20 40 80 160
[droperidol], µM
HCT116
Viability (%)
150 100 50 0
0 5 10 20 40 80
[ebastin], µM
HCT116
Viability (%)
150 100 50 0
0 5 10 20 40 80
[fluoxetine], µM
HCT116
Viability (%)
150 100 50 0
0 5 10 20 40 80
[fluspirilene], µM
HCT116
Viability (%)
150 100 50 0
0 10 20 40 80 160
[haloperidol], µM
HCT116
Viability (%)
150 100 50 0
0 10 20 40 80 160
[iloperidone], µM
HCT116
Viability (%)
150 100 50 0
0 10 20 40 80 160
[ketanserin], µM
HCT116
Viability (%)
150 100 50 0
0 10 20 40 80 160
[metoclopramide], µM
HCT116
Viability (%)
150 100 50 0
0 5 10 20 40 80
[nefazodone], µM
HCT116
Viability (%)
150 100 50 0
0 10 20 40 80 160
[paliperidone], µM
HCT116
Viability (%)
150 100 50 0
0 5 10 20 40 80
[penfluridol], µM
HCT116
Viability (%)
150 100 50 0
0 5 10 16 20 40 80
[pimozide], µM
HCT116
Viability (%)
150 100 50 0
0 10 20 40 80 160
[pipamperone], µM
HCT116
Viability (%)
150 100 50 0
0 10 20 40 80 160
[R59022], µM
HCT116
Viability (%)
150 100 50 0
0 10 20 40 80 160
[R59949], µM
HCT116
Viability (%)
150 100 50 0
0 10 20 40 80 160
[risperidone], µM
HCT116
Viability (%)
150 100 50 0
0 10 20 40 80 160
[ritanserin], µM
HCT116
Viability (%)
150 100 50 0
0 5 10 20 40 80
[spiperone], µM
HCT116
Viability (%)
150 100 50 0
0 10 20 40 80 160
[trazodone], µM
HCT116
Viability (%)
150 100 50 0
0 10 20 40 80 160
[urapidil], µM
HCT116
Viability (%)
150 100 50 0
0 10 20 40 80 160
[Way-100135], µM
HCT116
Viability (%)
150 100 50 0
0 10 20 40 80 160
[ziprazidone], µM

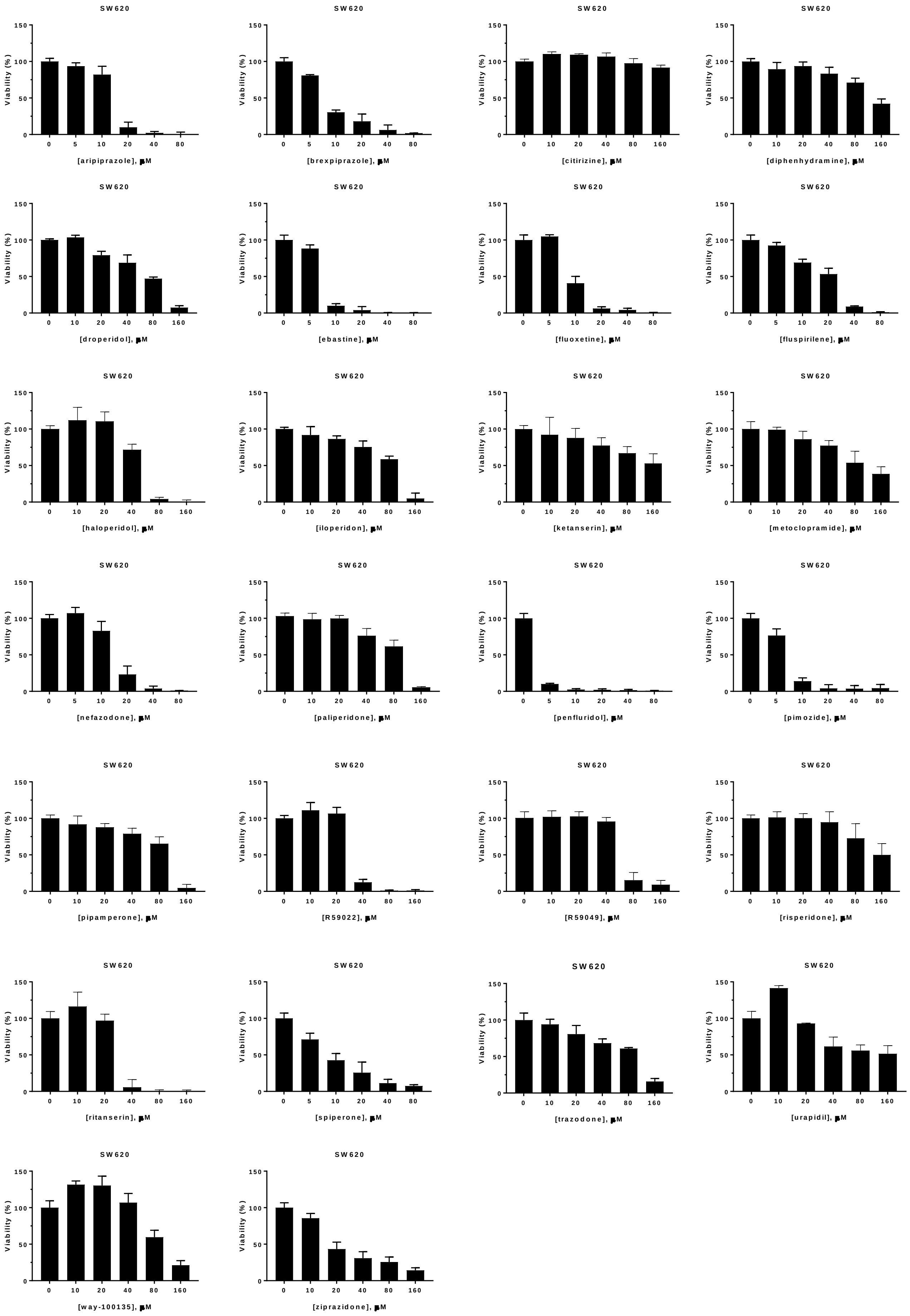
SW620
Viability (%)
[aripiprazole], µM
[brexpiprazole], µM
[citirizine], µM
[diphenhydramine], µM
[droperidol], µM
[ebastine], µM
[fluoxetine], µM
[fluspirilene], µM
[haloperidol], µM
[iloperidon], µM
[ketanserin], µM
[metoclopramide], µM
[nefazodone], µM
[paliperidone], µM
[penfluridol], µM
[pimozide], µM
[pipamperone], µM
[R59022], µM
[R59049], µM
[risperidone], µM
[ritanserin], µM
[spiperone], µM
[trazodone], µM
[urapidil], µM
[way-100135], µM
[ziprazidone], µM

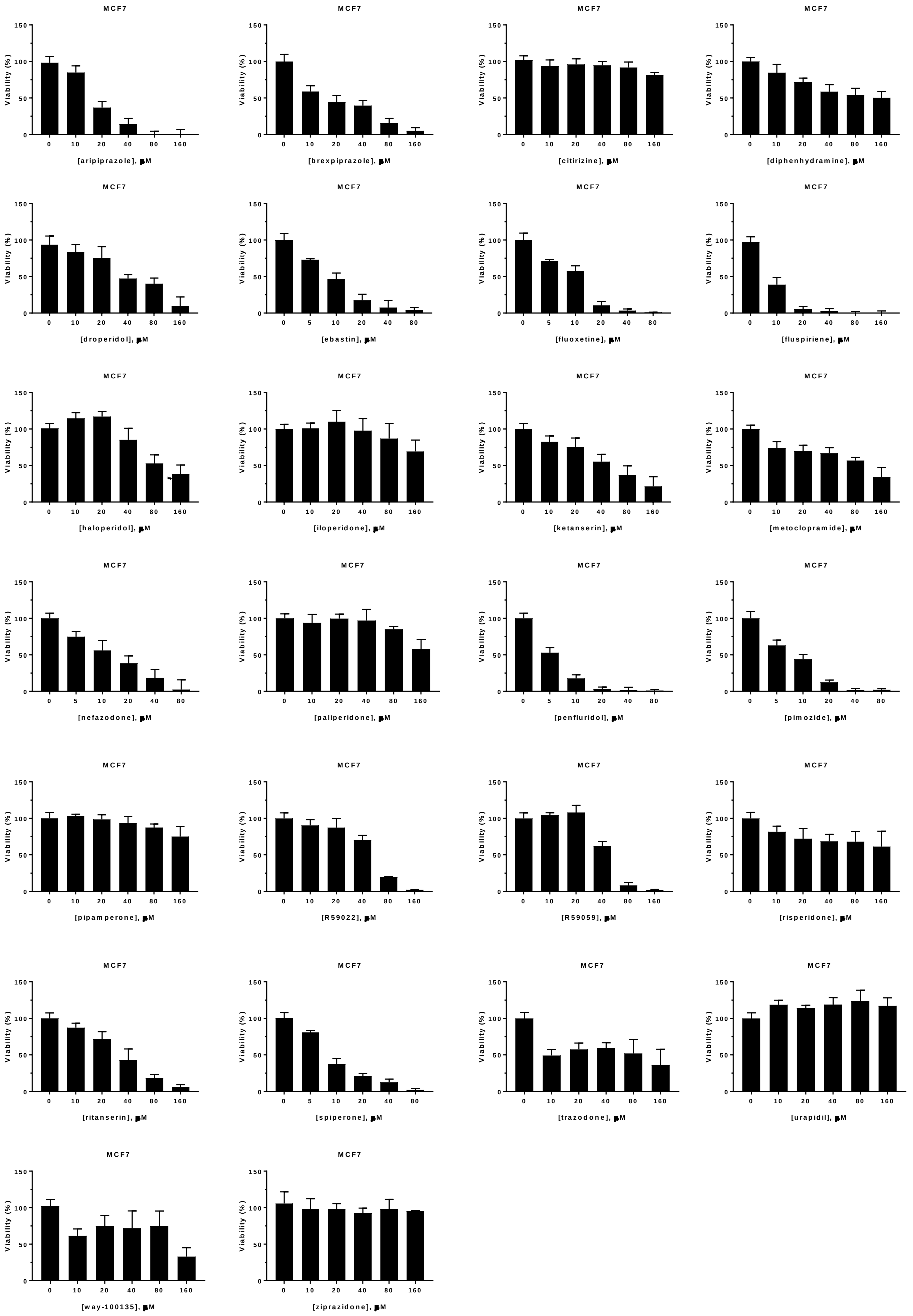
MCF7
Viability (%)
[aripiprazole], µM
MCF7
Viability (%)
[brexpiprazole], µM
MCF7
Viability (%)
[citirizine], µM
MCF7
Viability (%)
[diphenhydramine], µM
MCF7
Viability (%)
[droperidol], µM
MCF7
Viability (%)
[ebastin], µM
MCF7
Viability (%)
[fluoxetine], µM
MCF7
Viability (%)
[fluspiriene], µM
MCF7
Viability (%)
[haloperidol], µM
MCF7
Viability (%)
[iloperidone], µM
MCF7
Viability (%)
[ketanserin], µM
MCF7
Viability (%)
[metoclopramide], µM
MCF7
Viability (%)
[nefazodone], µM
MCF7
Viability (%)
[paliperidone], µM
MCF7
Viability (%)
[penfluridol], µM
MCF7
Viability (%)
[pimozide], µM
MCF7
Viability (%)
[pipamperone], µM
MCF7
Viability (%)
[R59022], µM
MCF7
Viability (%)
[R59059], µM
MCF7
Viability (%)
[risperidone], µM
MCF7
Viability (%)
[ritanserin], µM
MCF7
Viability (%)
[spiperone], µM
MCF7
Viability (%)
[trazodone], µM
MCF7
Viability (%)
[urapidil], µM
MCF7
Viability (%)
[way-100135], µM
MCF7
Viability (%)
[ziprazidone], µM

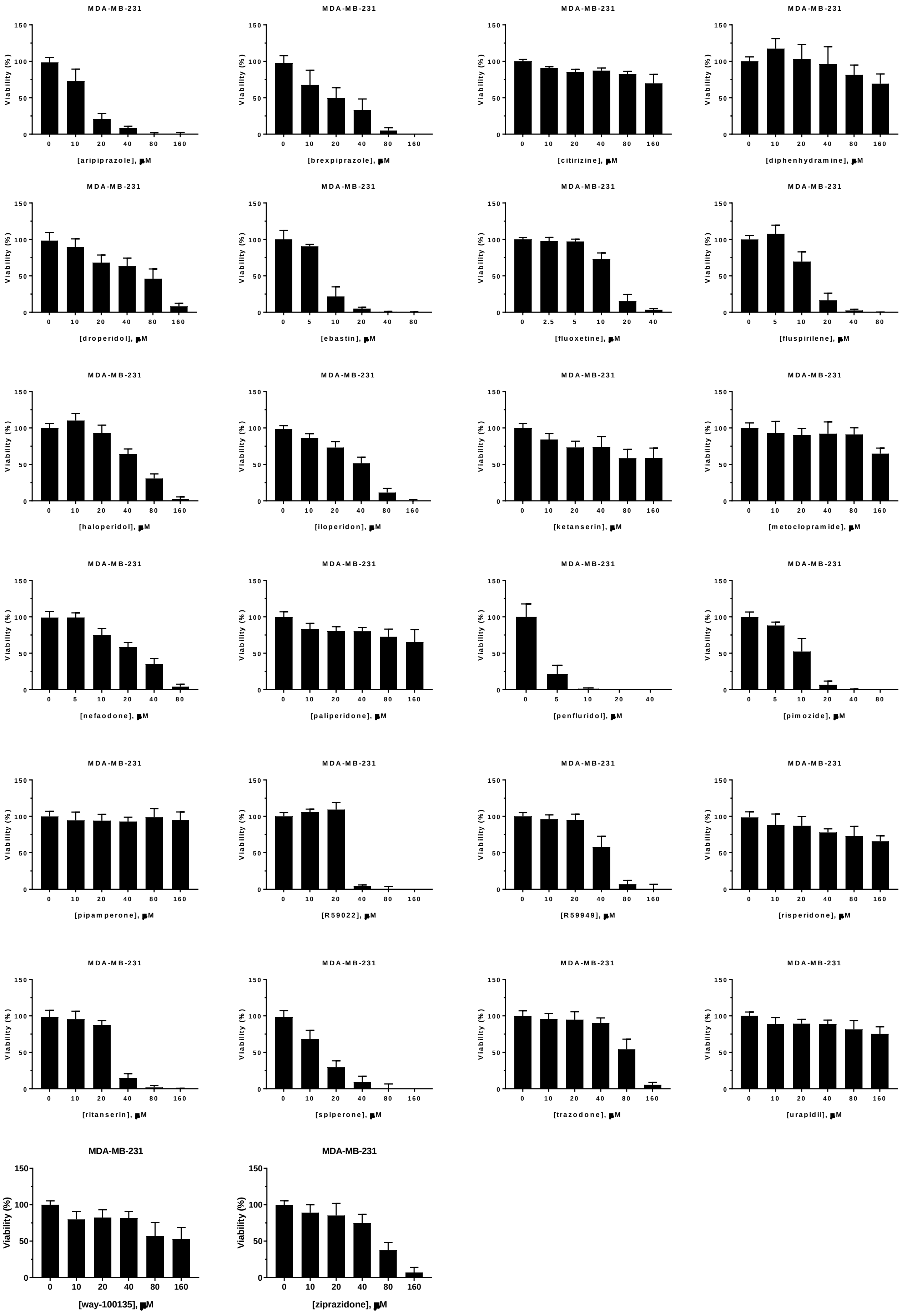
MDA-MB-231
Viability (%)
0 50 100 150
0 10 20 40 80 160
[aripiprazole], µM
[brexpiprazole], µM
[citirizine], µM
[diphenhydramine], µM
[droperidol], µM
0 5 10 20 40 80
[ebastin], µM
0 2.5 5 10 20 40
[fluoxetine], µM
[fluspirilene], µM
[haloperidol], µM
[iloperidon], µM
[ketanserin], µM
[metoclopramide], µM
[nefaodone], µM
[paliperidone], µM
0 5 10 20 40
[penfluridol], µM
[pimozide], µM
[pipamperone], µM
[R59022], µM
[R59949], µM
[risperidone], µM
[ritanserin], µM
[spiperone], µM
[trazodone], µM
[urapidil], µM
[way-100135], µM
[ziprazidone], µM

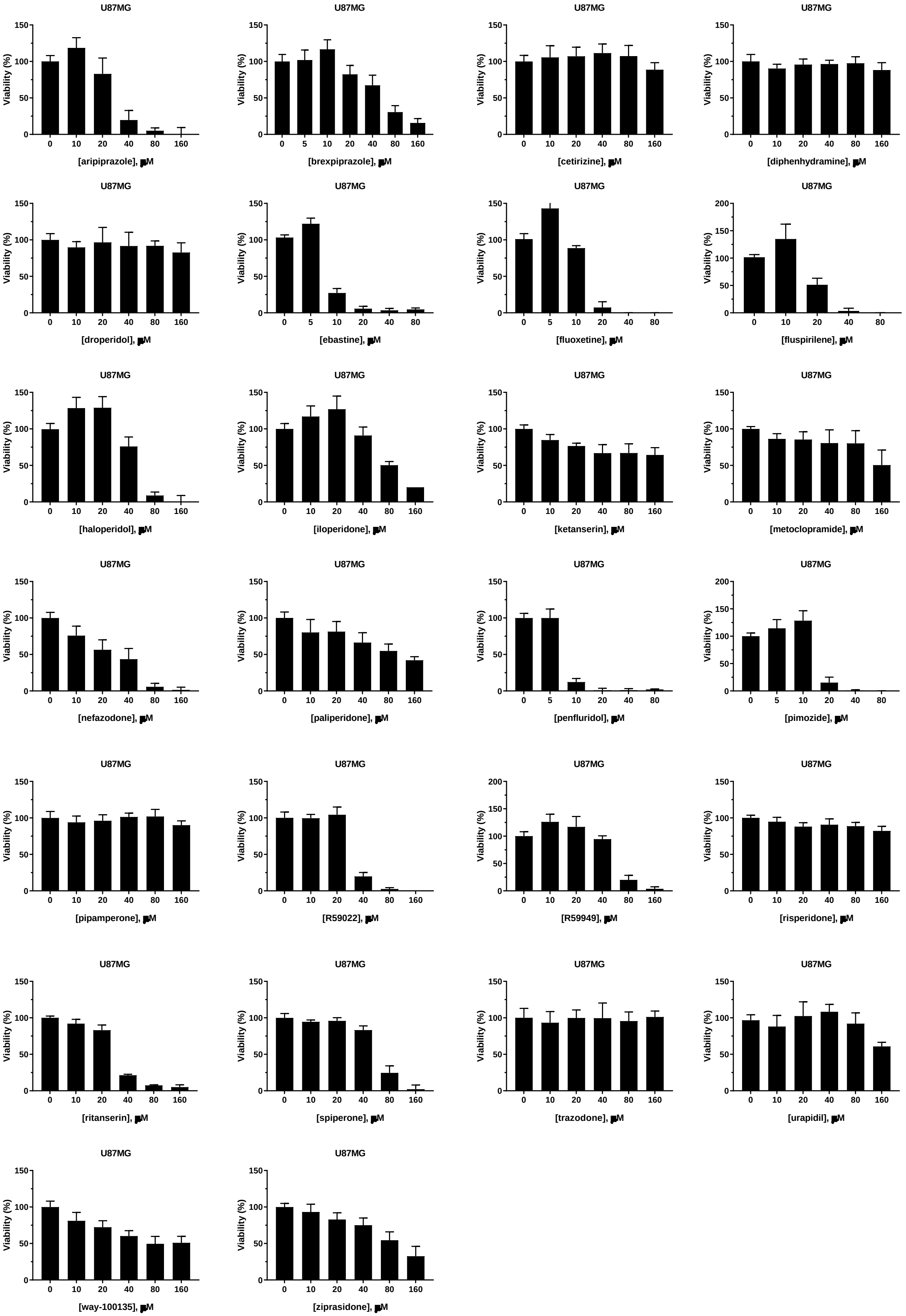
U87MG
Viability (%)
[aripiprazole], µM
U87MG
Viability (%)
[brexpiprazole], µM
U87MG
Viability (%)
[cetirizine], µM
U87MG
Viability (%)
[diphenhydramine], µM
U87MG
Viability (%)
[droperidol], µM
U87MG
Viability (%)
[ebastine], µM
U87MG
Viability (%)
[fluoxetine], µM
U87MG
Viability (%)
[fluspirilene], µM
U87MG
Viability (%)
[haloperidol], µM
U87MG
Viability (%)
[iloperidone], µM
U87MG
Viability (%)
[ketanserin], µM
U87MG
Viability (%)
[metoclopramide], µM
U87MG
Viability (%)
[nefazodone], µM
U87MG
Viability (%)
[paliperidone], µM
U87MG
Viability (%)
[penfluridol], µM
U87MG
Viability (%)
[pimozide], µM
U87MG
Viability (%)
[pipamperone], µM
U87MG
Viability (%)
[R59022], µM
U87MG
Viability (%)
[R59949], µM
U87MG
Viability (%)
[risperidone], µM
U87MG
Viability (%)
[ritanserin], µM
U87MG
Viability (%)
[spiperone], µM
U87MG
Viability (%)
[trazodone], µM
U87MG
Viability (%)
[urapidil], µM
U87MG
Viability (%)
[way-100135], µM
U87MG
Viability (%)
[ziprasidone], µM

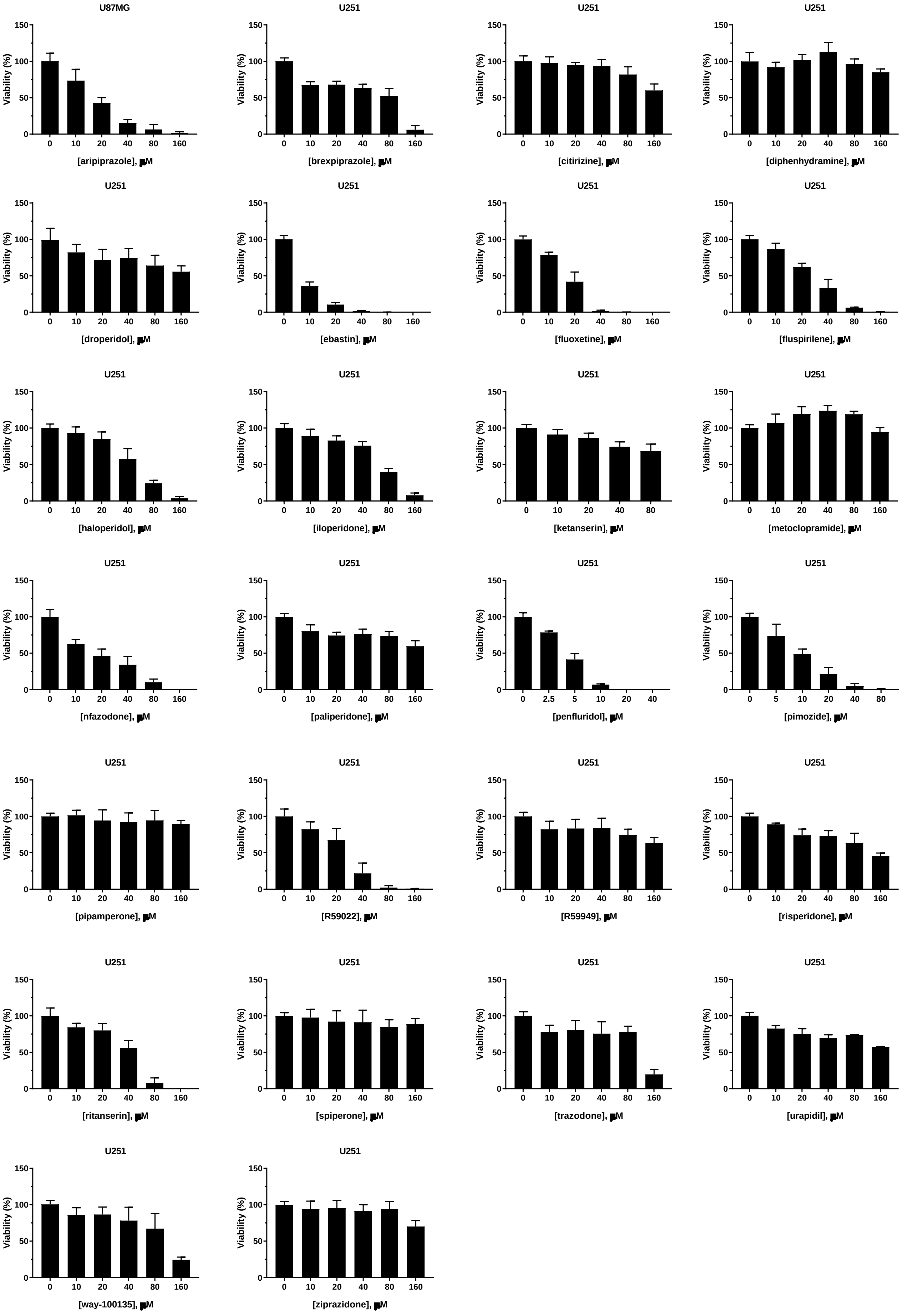

Supplementary figure 1. Effect of psychotropic drugs on cancer cell lines viability. Six cancer cell lines were treated for 72 h with scalar doses of drugs ranging from 10 to 160 µmol/L. The screened drugs included antipsychotics, antidepressant, antihistamines and three compounds used in scientific research with reported serotonin receptors antagonistic activity (R59949, R59022; WAY-100135). Cell viability is shown as percentage of viable cells versus control. Data are presented as mean ± standard deviation (SD) from three independent experiments, each performed in quadruplicate

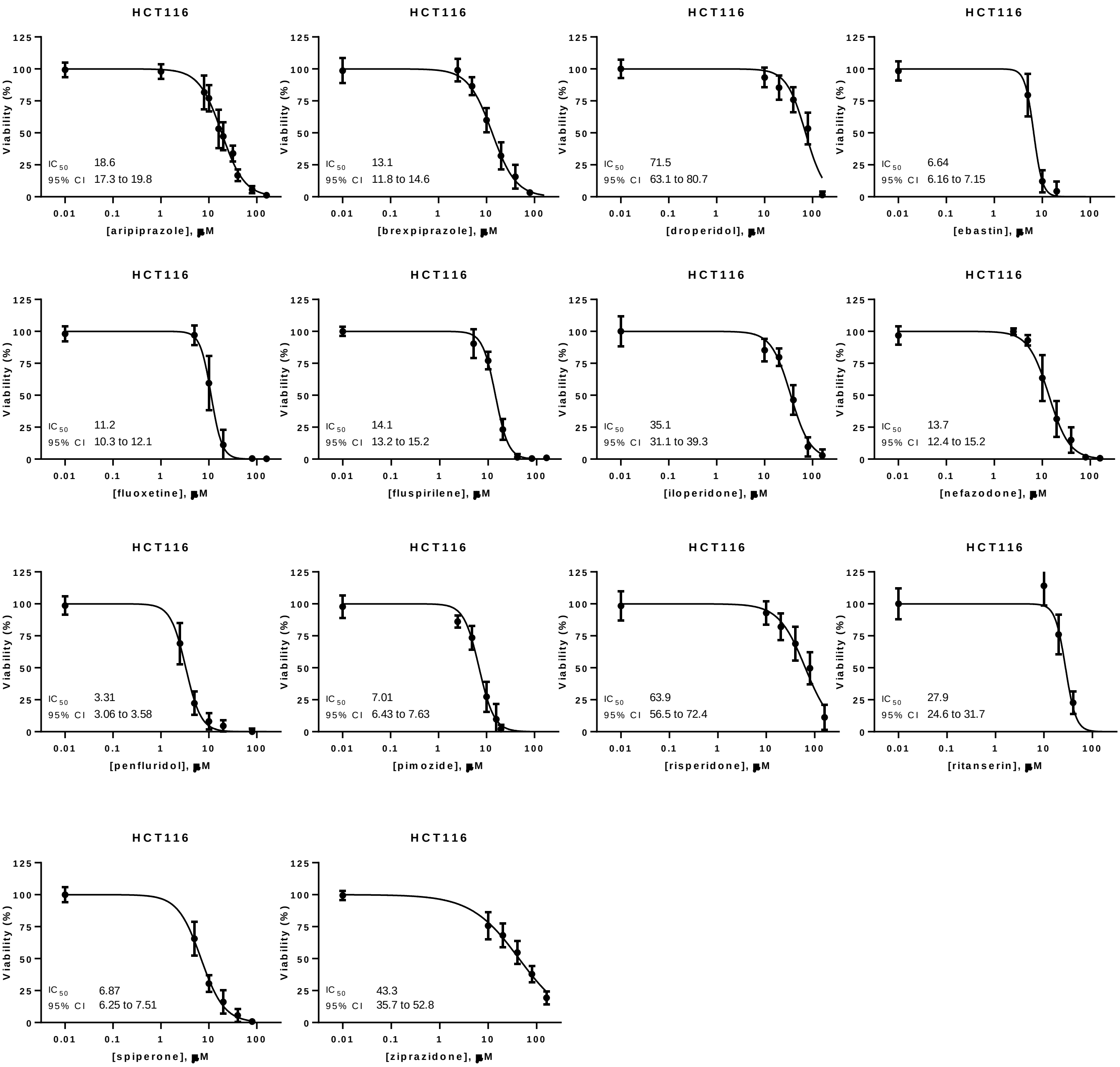
HCT116
Viability (%)
IC 50 18.6
95% CI 17.3 to 19.8
[aripiprazole], µM
HCT116
Viability (%)
IC 50 13.1
95% CI 11.8 to 14.6
[brexpiprazole], µM
HCT116
Viability (%)
IC 50 71.5
95% CI 63.1 to 80.7
[droperidol], µM
HCT116
Viability (%)
IC 50 6.64
95% CI 6.16 to 7.15
[ebastin], µM
HCT116
Viability (%)
IC 50 11.2
95% CI 10.3 to 12.1
[fluoxetine], µM
HCT116
Viability (%)
IC 50 14.1
95% CI 13.2 to 15.2
[fluspirilene], µM
HCT116
Viability (%)
IC 50 35.1
95% CI 31.1 to 39.3
[iloperidone], µM
HCT116
Viability (%)
IC 50 13.7
95% CI 12.4 to 15.2
[nefazodone], µM
HCT116
Viability (%)
IC 50 3.31
95% CI 3.06 to 3.58
[penfluridol], µM
HCT116
Viability (%)
IC 50 7.01
95% CI 6.43 to 7.63
[pimozide], µM
HCT116
Viability (%)
IC 50 63.9
95% CI 56.5 to 72.4
[risperidone], µM
HCT116
Viability (%)
IC 50 27.9
95% CI 24.6 to 31.7
[ritanserin], µM
HCT116
Viability (%)
IC 50 6.87
95% CI 6.25 to 7.51
[spiperone], µM
HCT116
Viability (%)
IC 50 43.3
95% CI 35.7 to 52.8
[ziprazidone], µM

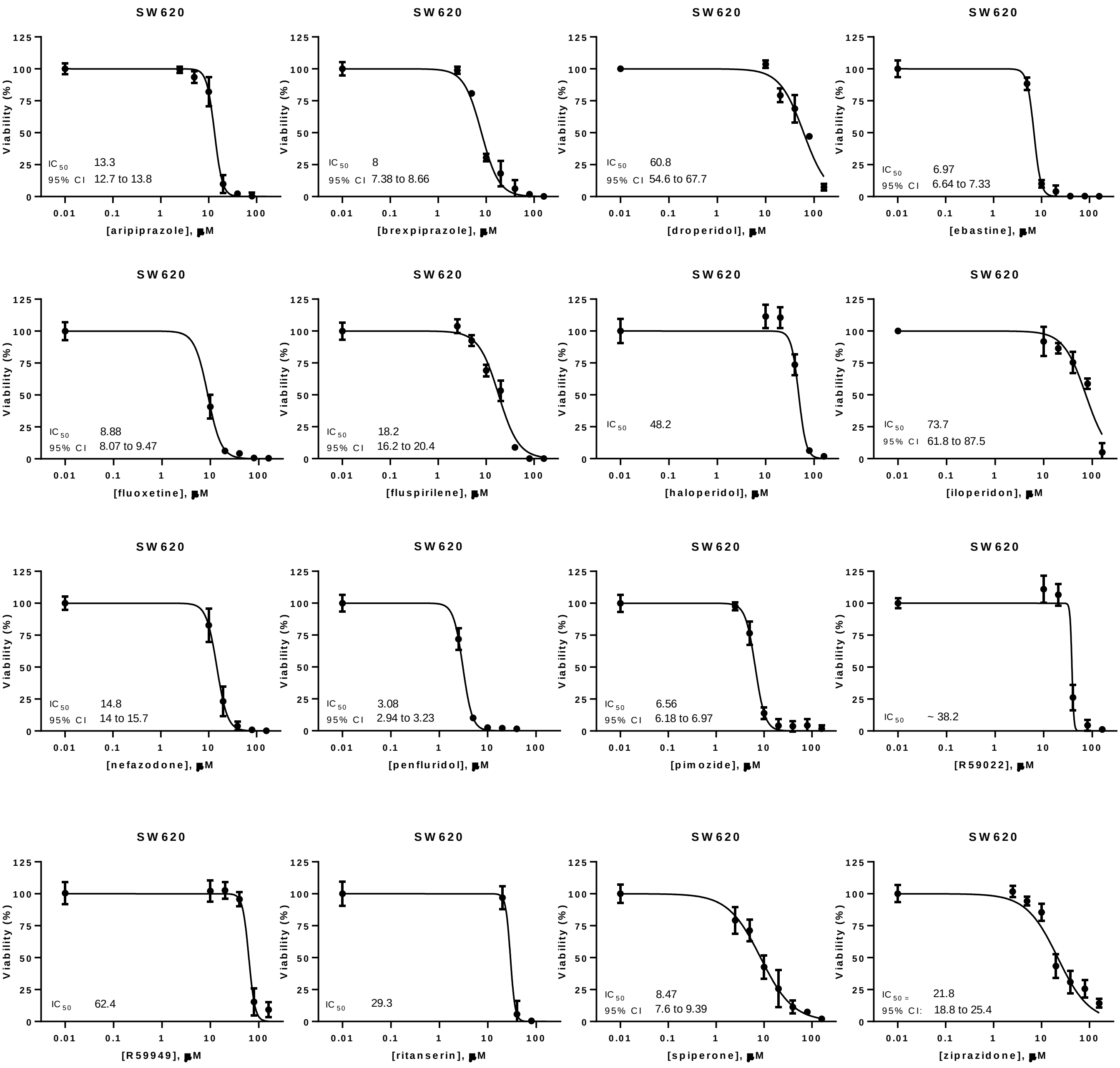
SW620
Viability (%)
IC 50 13.3
95% CI 12.7 to 13.8
[aripiprazole], µM
SW620
Viability (%)
IC 50 8
95% CI 7.38 to 8.66
[brexpiprazole], µM
SW620
Viability (%)
IC 50 60.8
95% CI 54.6 to 67.7
[droperidol], µM
SW620
Viability (%)
IC 50 6.97
95% CI 6.64 to 7.33
[ebastine], µM
SW620
Viability (%)
IC 50 8.88
95% CI 8.07 to 9.47
[fluoxetine], µM
SW620
Viability (%)
IC 50 18.2
95% CI 16.2 to 20.4
[fluspirilene], µM
SW620
Viability (%)
IC 50 48.2
[haloperidol], µM
SW620
Viability (%)
IC 50 73.7
95% CI 61.8 to 87.5
[iloperidon], µM
SW620
Viability (%)
IC 50 14.8
95% CI 14 to 15.7
[nefazodone], µM
SW620
Viability (%)
IC 50 3.08
95% CI 2.94 to 3.23
[penfluridol], µM
SW620
Viability (%)
IC 50 6.56
95% CI 6.18 to 6.97
[pimozide], µM
SW620
Viability (%)
IC 50 ~ 38.2
[R59022], µM
SW620
Viability (%)
IC 50 62.4
[R59949], µM
SW620
Viability (%)
IC 50 29.3
[ritanserin], µM
SW620
Viability (%)
IC 50 8.47
95% CI 7.6 to 9.39
[spiperone], µM
SW620
Viability (%)
IC 50 = 21.8
95% CI: 18.8 to 25.4
[ziprazidone], µM

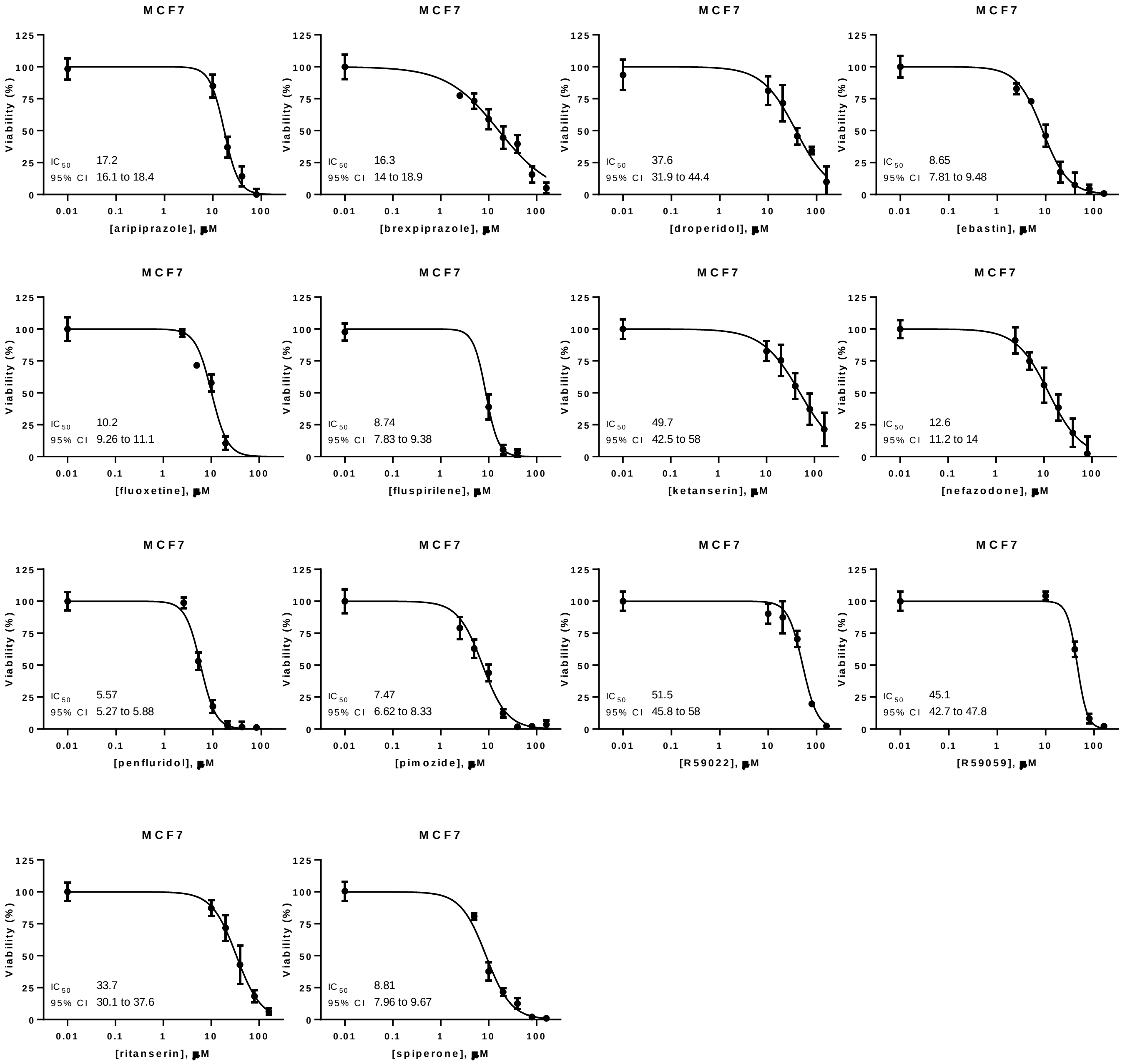
MCF7
Viability (%)
IC50 17.2
95% CI 16.1 to 18.4
[aripiprazole], µM
MCF7
Viability (%)
IC50 16.3
95% CI 14 to 18.9
[brexpiprazole], µM
MCF7
Viability (%)
IC50 37.6
95% CI 31.9 to 44.4
[droperidol], µM
MCF7
Viability (%)
IC50 8.65
95% CI 7.81 to 9.48
[ebastin], µM
MCF7
Viability (%)
IC50 10.2
95% CI 9.26 to 11.1
[fluoxetine], µM
MCF7
Viability (%)
IC50 8.74
95% CI 7.83 to 9.38
[fluspirilene], µM
MCF7
Viability (%)
IC50 49.7
95% CI 42.5 to 58
[ketanserin], µM
MCF7
Viability (%)
IC50 12.6
95% CI 11.2 to 14
[nefazodone], µM
MCF7
Viability (%)
IC50 5.57
95% CI 5.27 to 5.88
[penfluridol], µM
MCF7
Viability (%)
IC50 7.47
95% CI 6.62 to 8.33
[pimozide], µM
MCF7
Viability (%)
IC50 51.5
95% CI 45.8 to 58
[R59022], µM
MCF7
Viability (%)
IC50 45.1
95% CI 42.7 to 47.8
[R59059], µM
MCF7
Viability (%)
IC50 33.7
95% CI 30.1 to 37.6
[ritanserin], µM
MCF7
Viability (%)
IC50 8.81
95% CI 7.96 to 9.67
[spiperone], µM

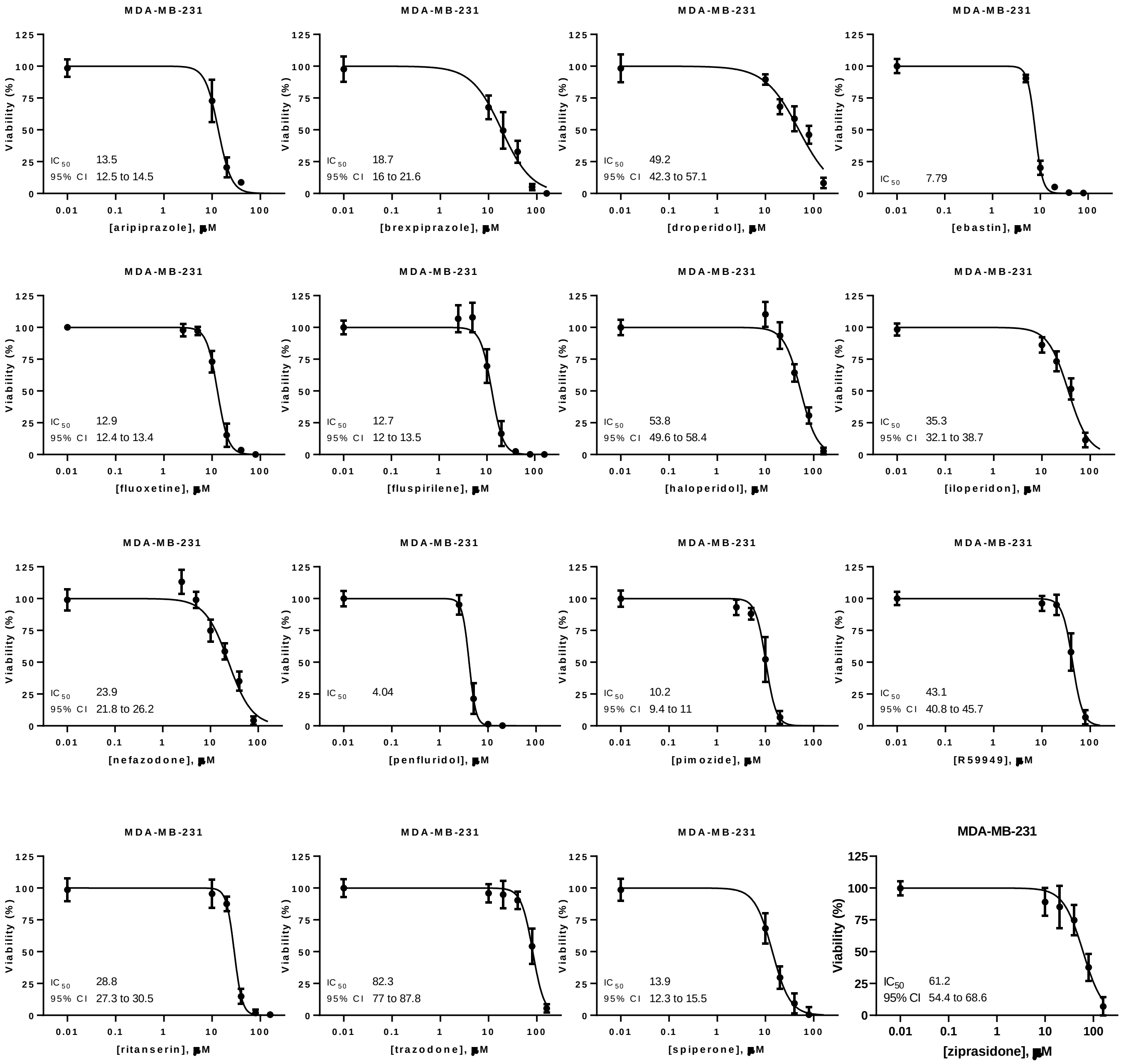

MDA-MB-231
Viability (%)
IC50 13.5
95% CI 12.5 to 14.5
[aripiprazole], µM
MDA-MB-231
Viability (%)
IC50 18.7
95% CI 16 to 21.6
[brexpiprazole], µM
MDA-MB-231
Viability (%)
IC50 49.2
95% CI 42.3 to 57.1
[droperidol], µM
MDA-MB-231
Viability (%)
IC50 7.79
[ebastin], µM
MDA-MB-231
Viability (%)
IC50 12.9
95% CI 12.4 to 13.4
[fluoxetine], µM
MDA-MB-231
Viability (%)
IC50 12.7
95% CI 12 to 13.5
[fluspirilene], µM
MDA-MB-231
Viability (%)
IC50 53.8
95% CI 49.6 to 58.4
[haloperidol], µM
MDA-MB-231
Viability (%)
IC50 35.3
95% CI 32.1 to 38.7
[iloperidon], µM
MDA-MB-231
Viability (%)
IC50 23.9
95% CI 21.8 to 26.2
[nefazodone], µM
MDA-MB-231
Viability (%)
IC50 4.04
[penfluridol], µM
MDA-MB-231
Viability (%)
IC50 10.2
95% CI 9.4 to 11
[pimozide], µM
MDA-MB-231
Viability (%)
IC50 43.1
95% CI 40.8 to 45.7
[R59949], µM
MDA-MB-231
Viability (%)
IC50 28.8
95% CI 27.3 to 30.5
[ritanserin], µM
MDA-MB-231
Viability (%)
IC50 82.3
95% CI 77 to 87.8
[trazodone], µM
MDA-MB-231
Viability (%)
IC50 13.9
95% CI 12.3 to 15.5
[spiperone], µM
MDA-MB-231
Viability (%)
IC50 61.2
95% CI 54.4 to 68.6
[ziprasidone], µM

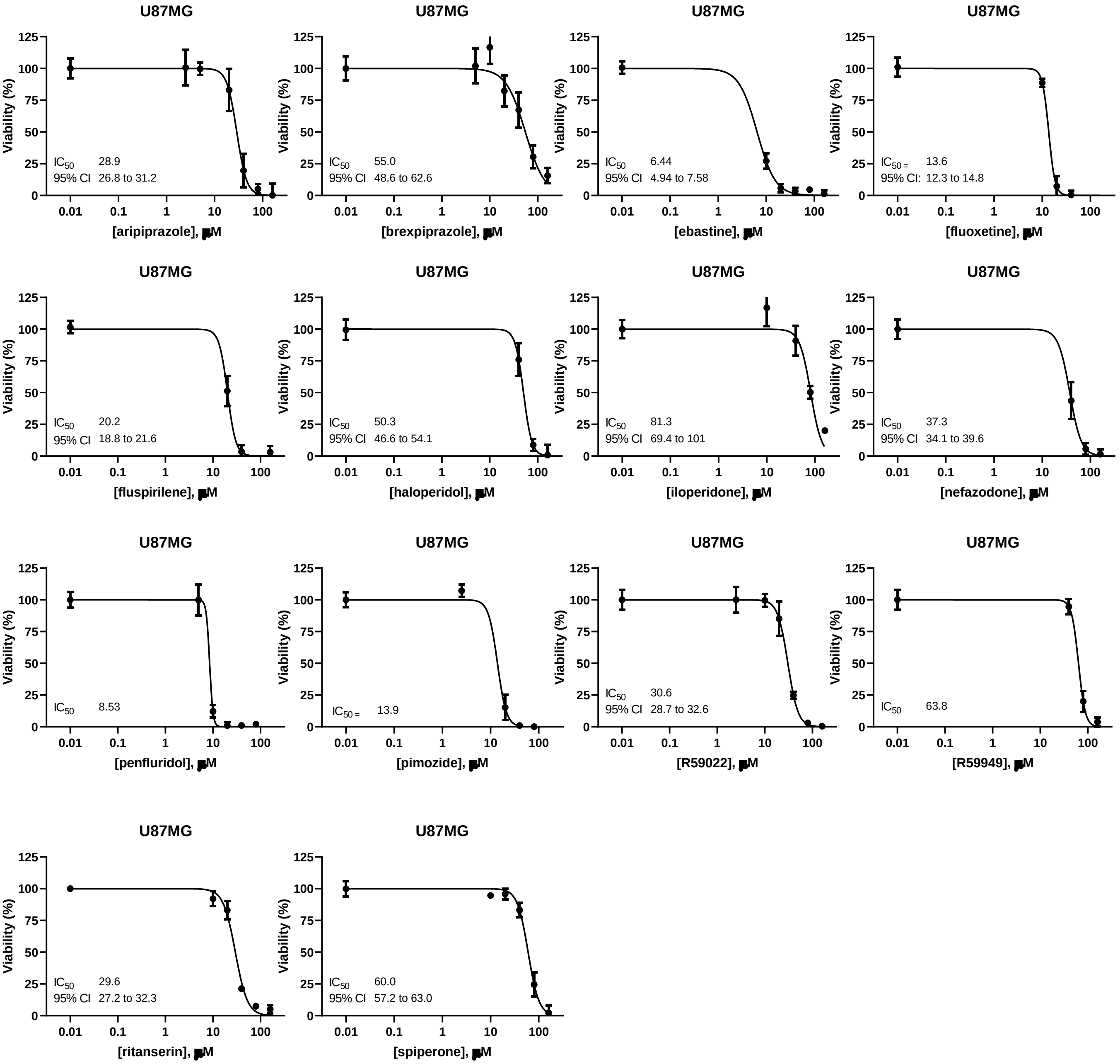
U87MG
Viability (%)
IC50 28.9
95% CI 26.8 to 31.2
[aripiprazole], μM
U87MG
Viability (%)
IC50 55.0
95% CI 48.6 to 62.6
[brexpiprazole], μM
U87MG
Viability (%)
IC50 6.44
95% CI 4.94 to 7.58
[ebastine], μM
U87MG
Viability (%)
IC50 = 13.6
95% CI: 12.3 to 14.8
[fluoxetine], μM
U87MG
Viability (%)
IC50 20.2
95% CI 18.8 to 21.6
[fluspirilene], μM
U87MG
Viability (%)
IC50 50.3
95% CI 46.6 to 54.1
[haloperidol], μM
U87MG
Viability (%)
IC50 81.3
95% CI 69.4 to 101
[iloperidone], μM
U87MG
Viability (%)
IC50 37.3
95% CI 34.1 to 39.6
[nefazodone], μM
U87MG
Viability (%)
IC50 8.53
[penfluridol], μM
U87MG
Viability (%)
IC50 = 13.9
[pimozide], μM
U87MG
Viability (%)
IC50 30.6
95% CI 28.7 to 32.6
[R59022], μM
U87MG
Viability (%)
IC50 63.8
[R59949], μM
U87MG
Viability (%)
IC50 29.6
95% CI 27.2 to 32.3
[ritanserin], μM
U87MG
Viability (%)
IC50 60.0
95% CI 57.2 to 63.0
[spiperone], μM

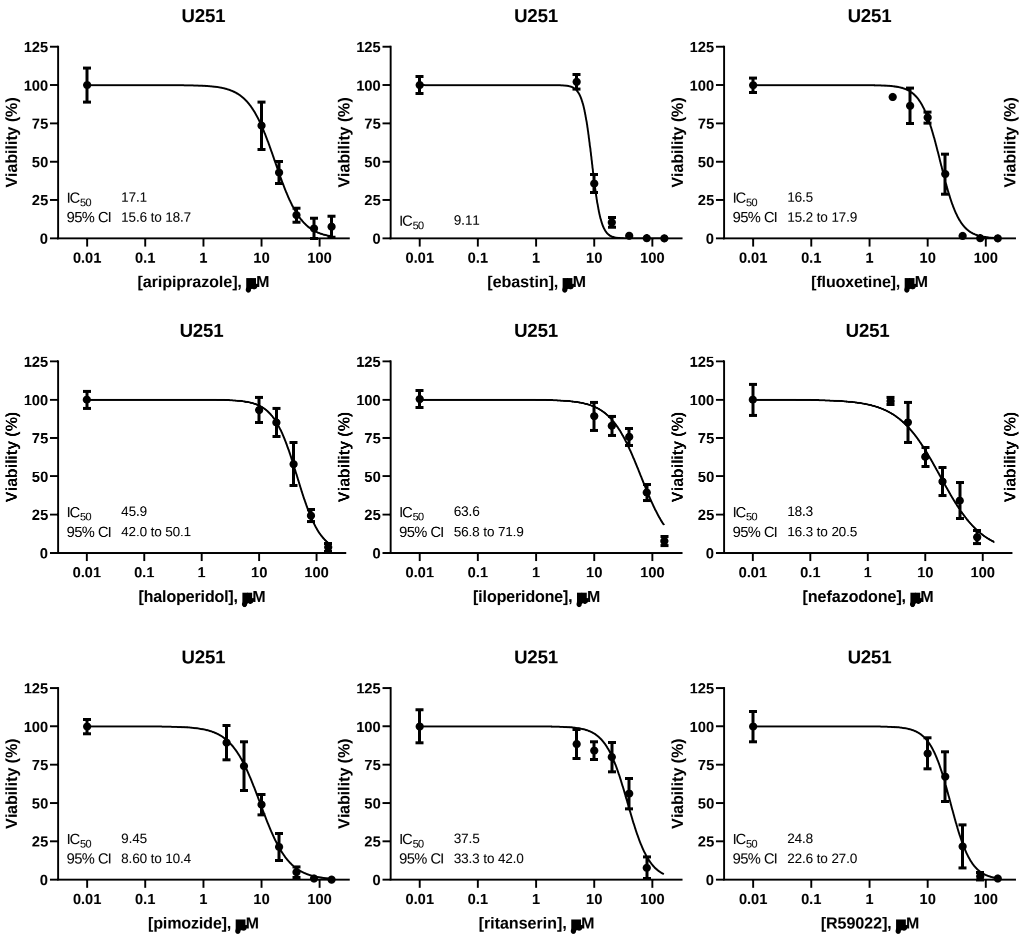

Supplementary figure 2. Dose response curves showing the cytotoxic effect of psychotropic drugs on neoplastic cells. Cells were treated for 72 h with scalar doses of drugs ranging from 10 to 160 μmol/L. Viabilities were assessed by MTT assay, normalized for cells treated with vehicle only and expressed as percentage. The best fit values of IC_{50} values were calculated the by using a variable slope model (GraphPad Prism 7). Each data point represents the mean of at least three independent experiments. IC_{50}, drug concentration reducing by 50% viability compared to control; 95% CI, 95% confidence interval.

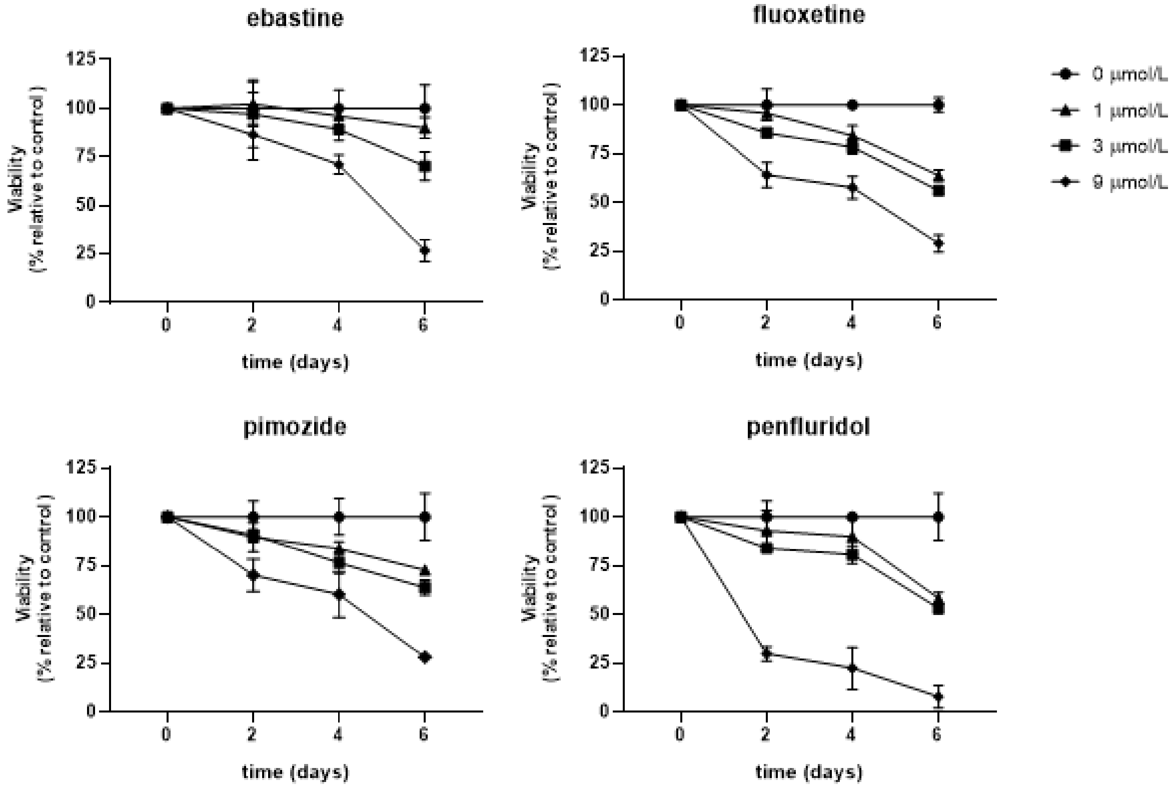

Supplementary Figure 3. Dose and time response curves showing the cytotoxic effect of ebastine, fluoxetine, pimozide and penfluridol on MCF7 cells. Cell were treated for 6 days with increasing doses of drugs. Viabilities were assessed by MTT assay and normalized relative to controls treated with vehicle only. Data are expressed as the mean ± SD of one representative out of three independent experiments performed in quintuplicate.3

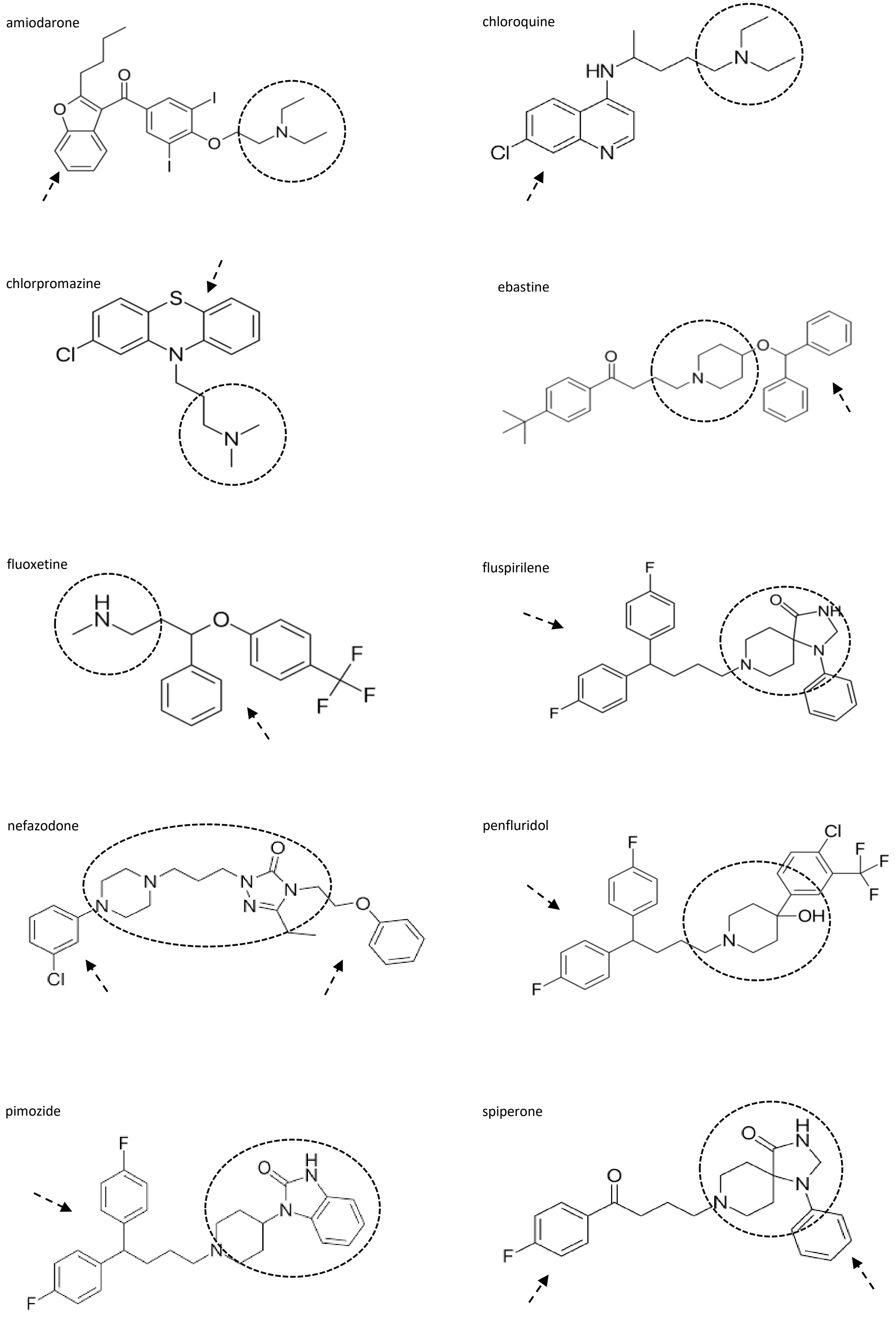

Supplementary Figure 4. CADs features of psychotropic drugs with antitumoral activity. Structure of cationic amphiphilic drugs and psychotropic drugs are shown. Circles indicate the hydrophilic region containing an amine group protonated at physiological pH, arrows indicate the hydrophobic region containing aromatic groups.

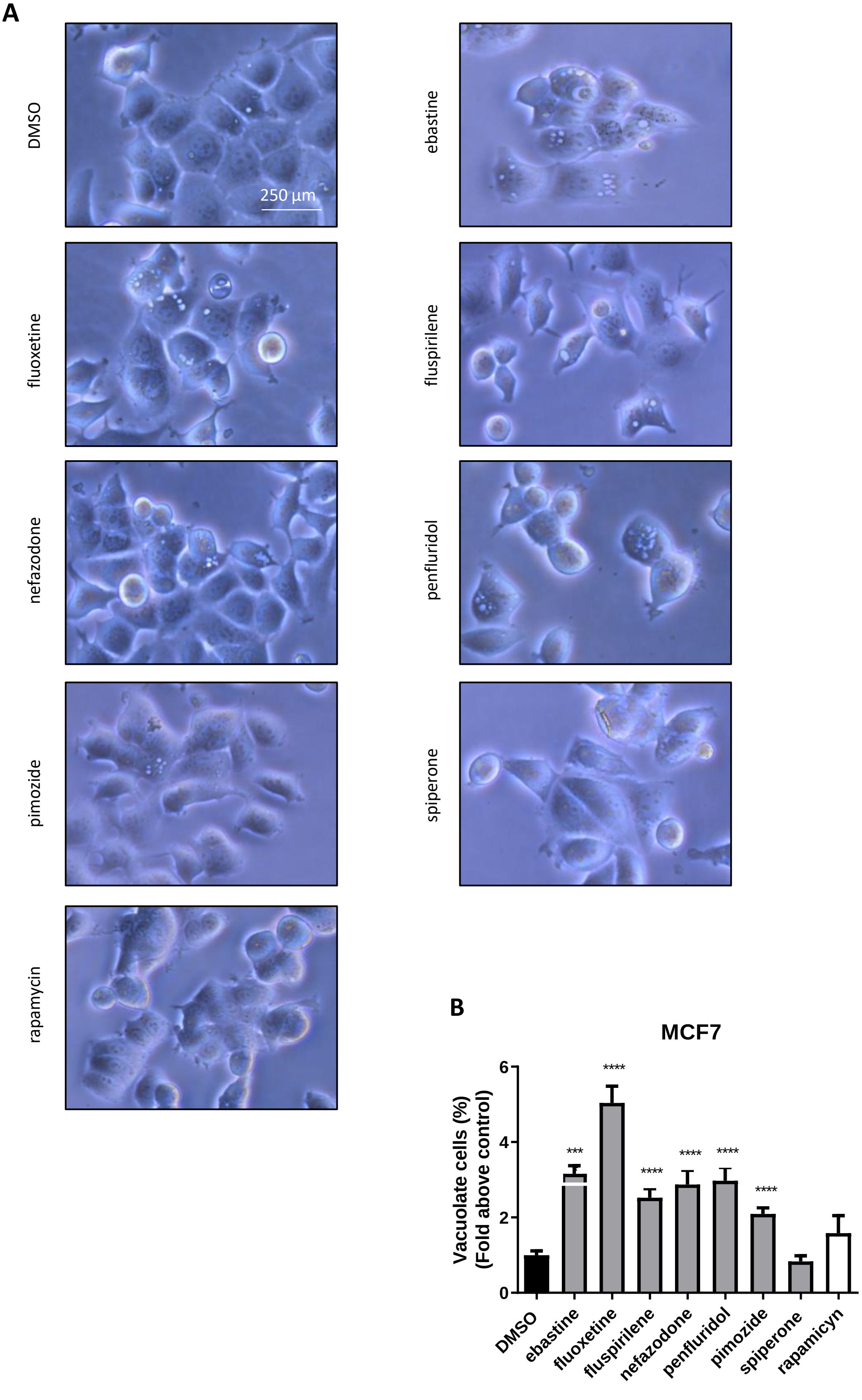

Supplementary Figure 5. Vacuolar structures formation after treatment of MCF7 cells with psychotropic drugs. Morphological alterations associated with the treatment of MCF7 cells with psychotropic drugs (5 µmol/L) were investigated after 6 h of exposure by phase contrast microscopy (original magnification 20x). Representative images of cells treated with DMSO, positive control, ebastine, fluoxetine, fluspirilene, nefazodone, penfluridol, pimozide, spiperone and rapamycin, positive control (A). Histogram showing quantification of vacuoles as fold change relative to control (B). Data are expressed as the mean ± SD of a representative experiment out of three independent experiments performed in triplicate. **, Student's T-test $p < 0.01$; ***, Student's T-test $p < 0.001$.

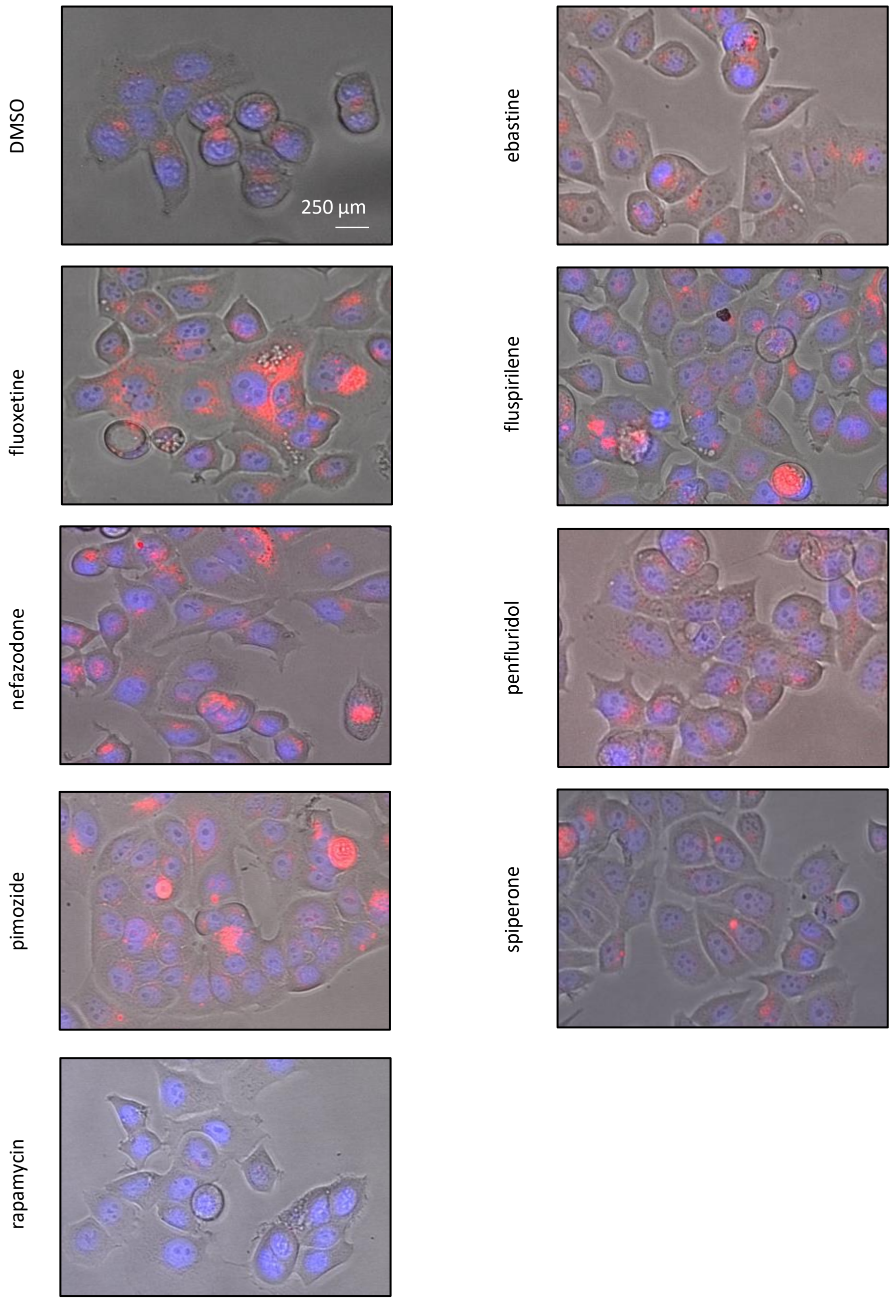

Supplementary Figure 6. Psychotropic drugs induce acidic compartment formation perturbing lysosomal and autophagic functioning. Vacuolar structures associated to treatment with psychotropic drugs in MCF7 were analyzed, after 6h exposure, staining cells with Lysotracker Deep Red. Pictures were acquired at fluorescence microscopy (magnification: 20x). representative images of cells treated with DMSO, negative control, ebastine, fluoxetine, fluspirilene, nefazodone, penfluridol, pimozide, spiperone and rapamycin

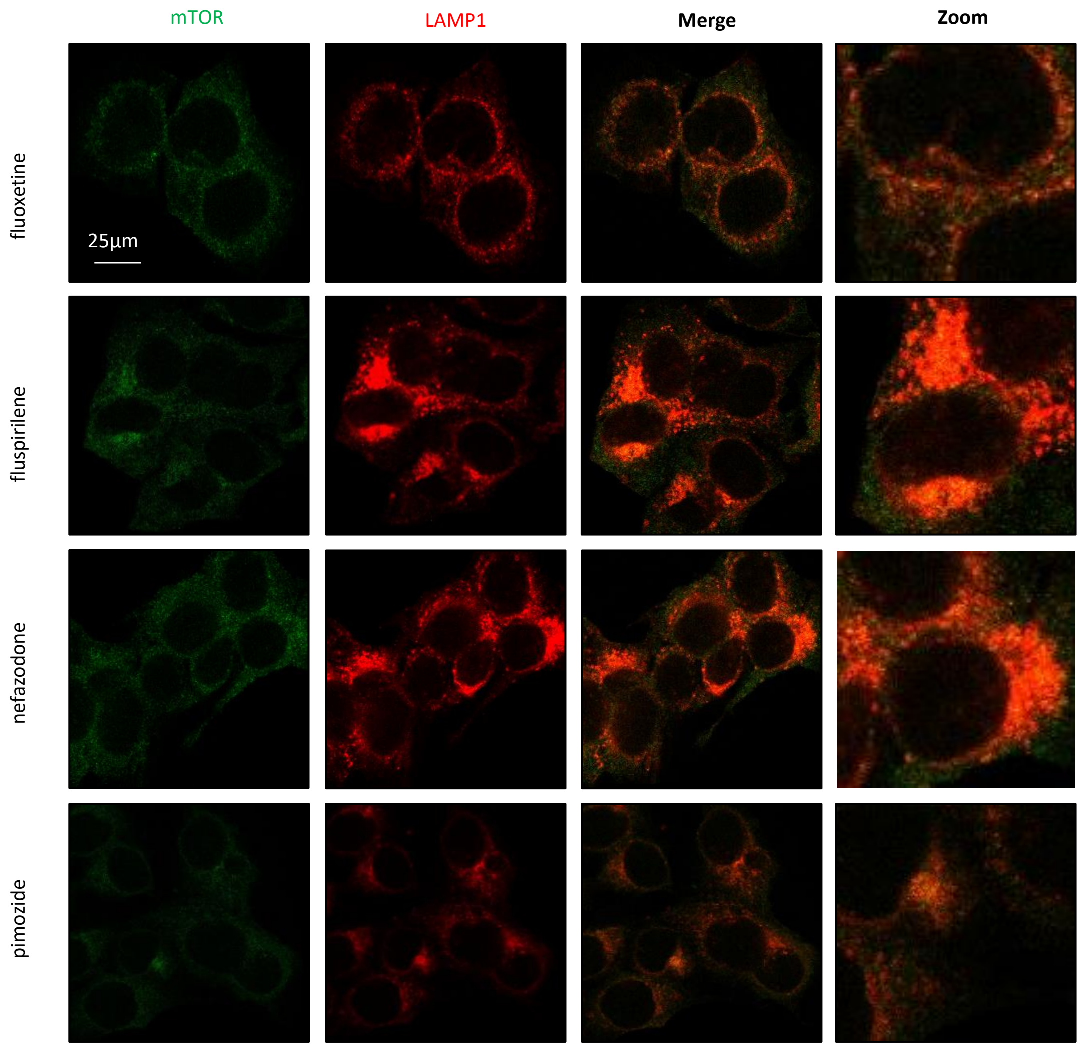

Supplementary Figure 7. Psychotropic drugs alter autophagy flux through mTOR pathway inhibition. Delocalization of mTOR from lysosomal membrane was evaluated in MCF7 after 16h treatment with psychoactive drugs. mTOR was stained using mTOR primary antibody and Alexa Fluor 488 secondary antibody (green). Lysosomes were stained using LAMP1 primary antibody and Alexa Fluor 536 secondary antibody (red). Representative images of fluoxetine, fluspirilene, nefazodone, pimozide

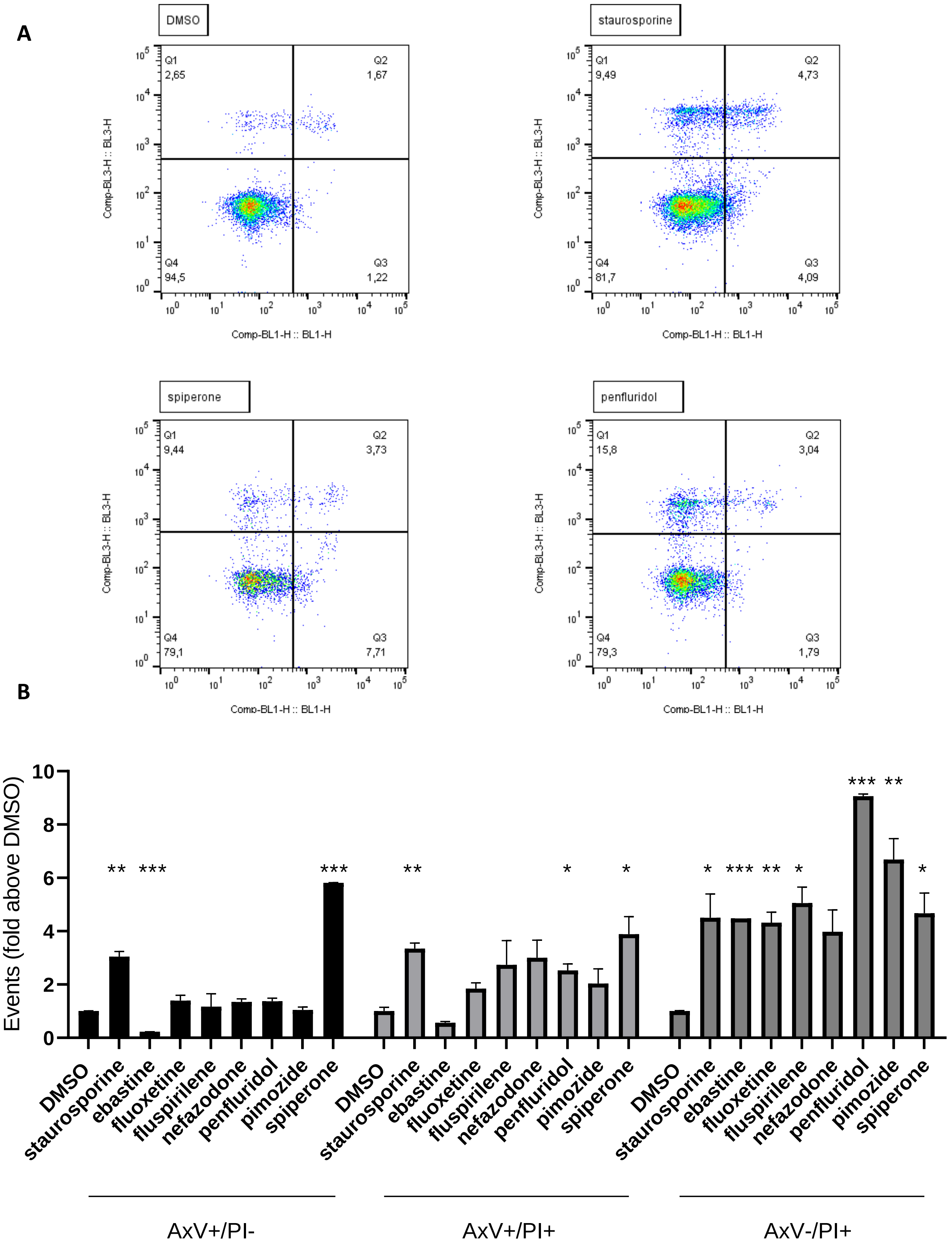

Supplementary Figure 8. Spiperone induces apoptosis in MCF7 cells. MCF7 cells treated for 48 hours with vehicle and psychotropic drugs at a concentration of 10 µmol/L for all the drugs except for penfluridol, 5 µmol/L. Staurosporine 0.5 µmol/L was used as apoptosis positive control. (A) representative populations observed by FACS analysis. (B) Graph showing Annexin V and Propidium Iodide positive/negative events as fold change relative to vehicle (DMSO). Data show mean ± SD of one representative experiment out of three independent experiments performed in duplicate. *, Student's T-test $p < 0.05$; **, Student's T-test $p < 0.01$; ***, Student's T-test $p < 0.001$

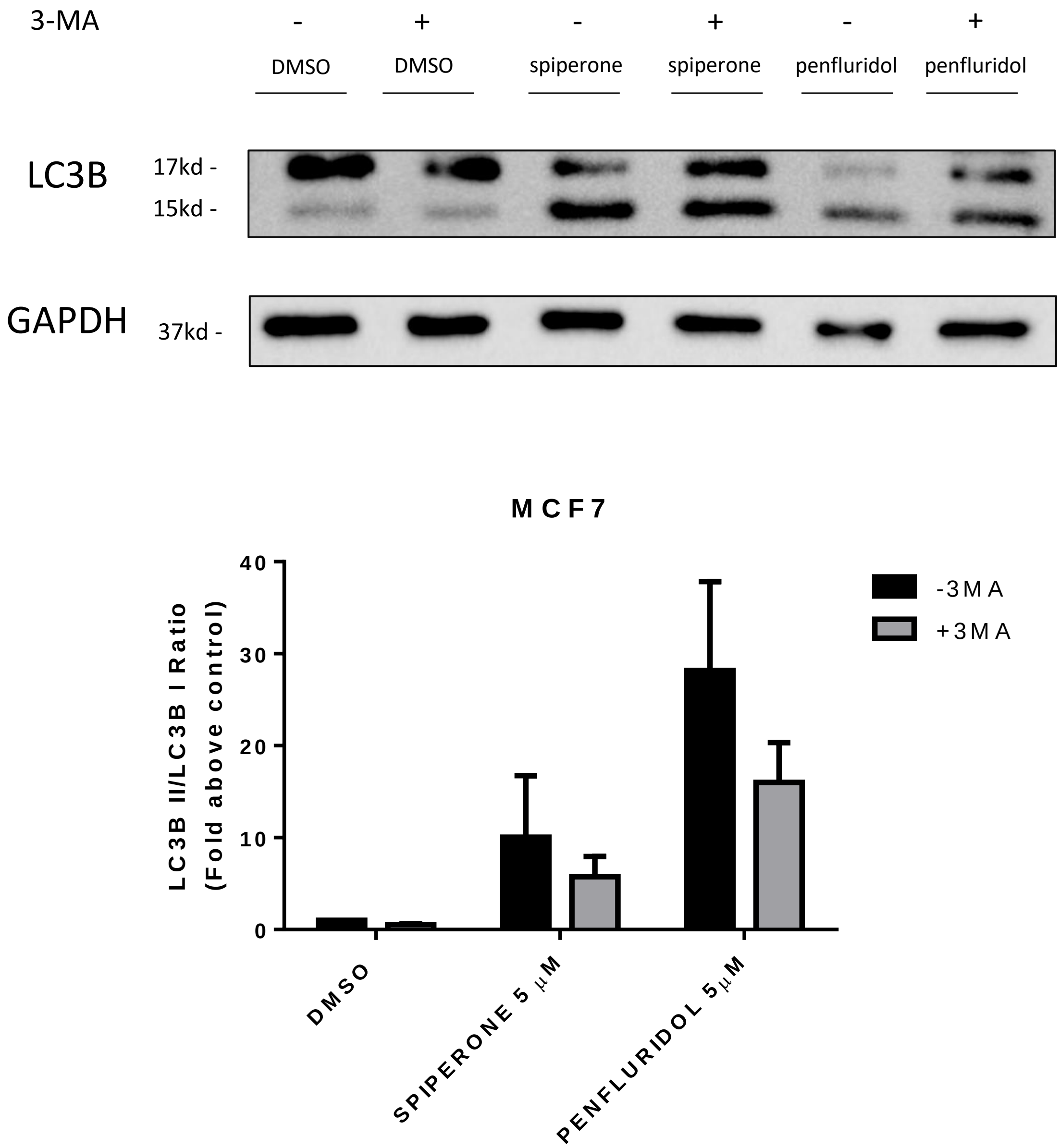

Supplementary Figure 9. 3-MA does not induce LC3B expression upon psychotropic drugs stimulation. Western blot analysis showing effects on autophagic pathway after 16 hours treatment with spiperone and penfluridol plus 3-MA on MCF7. Lysates analyzed LC3B and GAPDH (A). Histogram showing quantification of the relative expression of LC3B II/I. Densitometry are expressed as the mean ± SD of three independent experiments.

Raw data of western blot for review

P-AMPK alpha

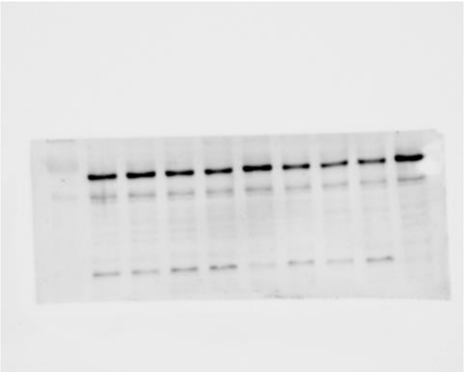

AMPK alpha

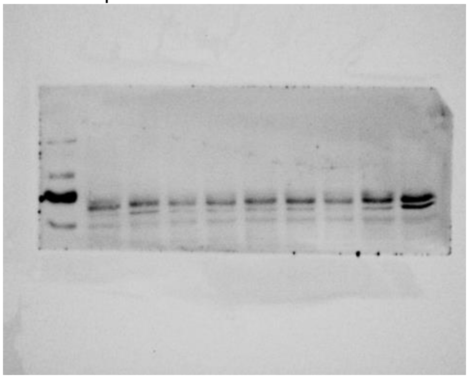

P-P70S6K

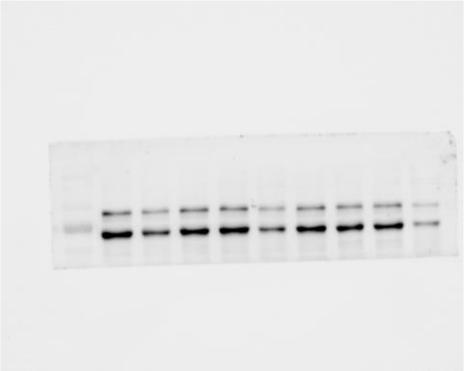

P70S6K

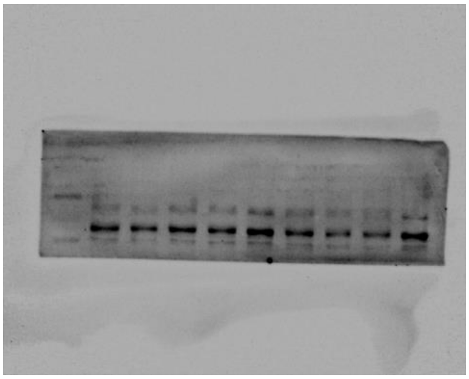

P-S6

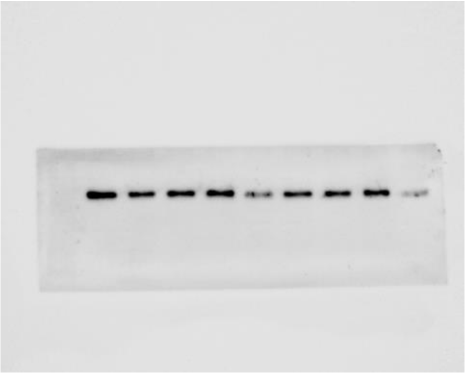

S6

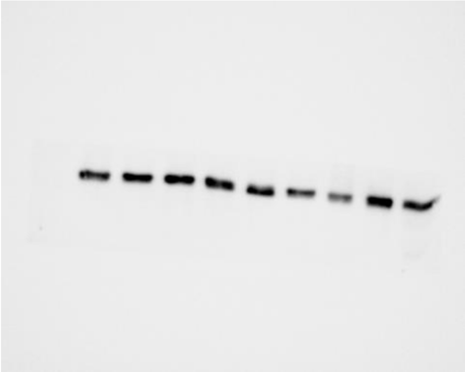

GAPDH

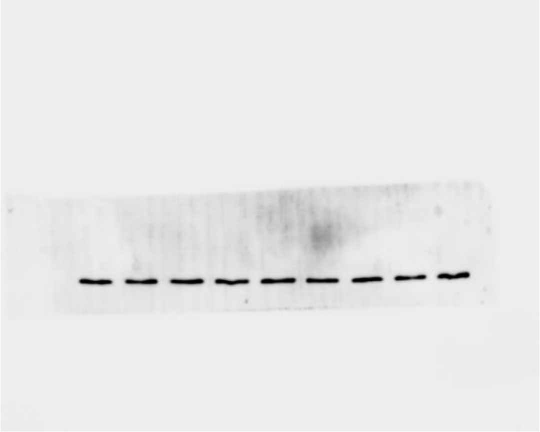

Figure 8. Psychotropic drugs alter autophagy flux through mTOR pathway inhibition. Western blot analysis showing effects on mTOR pathway after 16h treatment with psychotropic drugs on MCF7. Lysates analysed for p-AMPKα T172, AMPK, p-P70S6K T389, P70S6K, P-S6 S235/236, S6, LC3B and GAPDH (**A**). Histogram showing quantification of P70S6K (**B**), S6 (**C**) and AMPK (**D)** phosphorylation normalized on total protein P70S6K and S6. **Raw data of experiment 1**

P-AMPK alpha

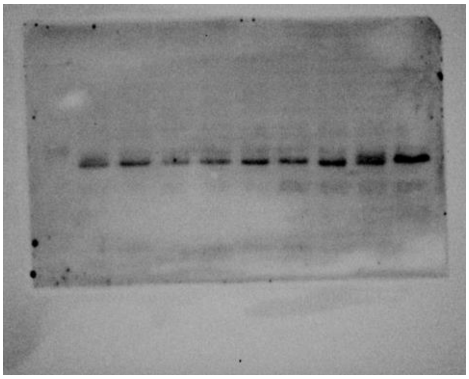

AMPK alpha

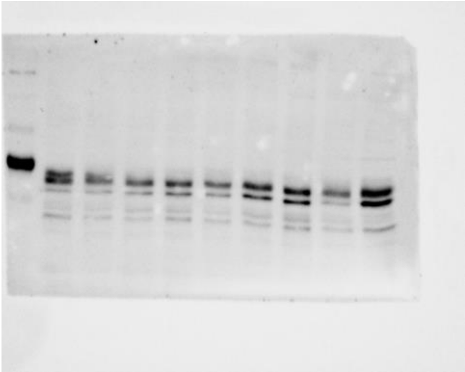

P-P70S6K

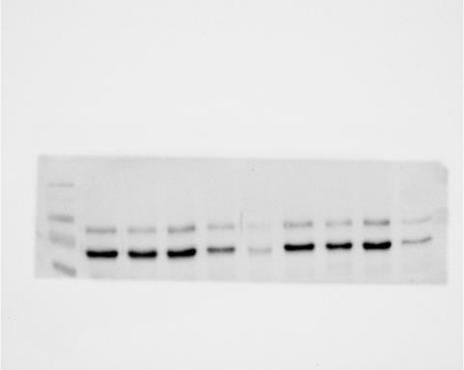

P70S6K

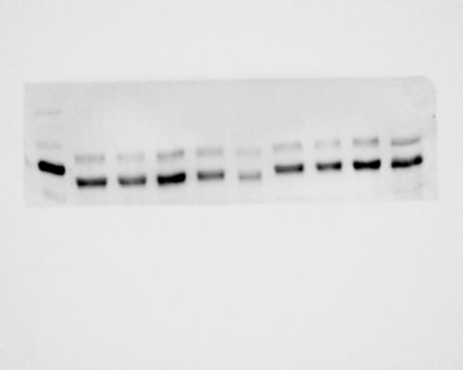

P-S6

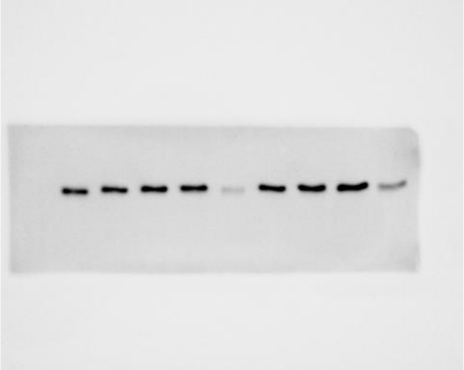

S6

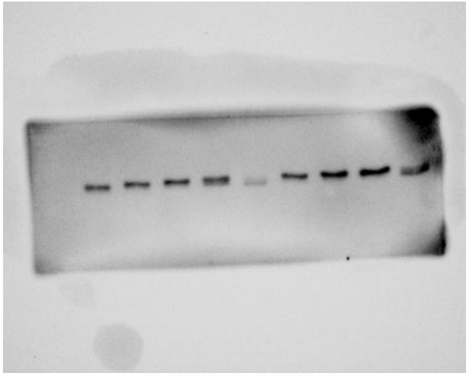

GAPDH

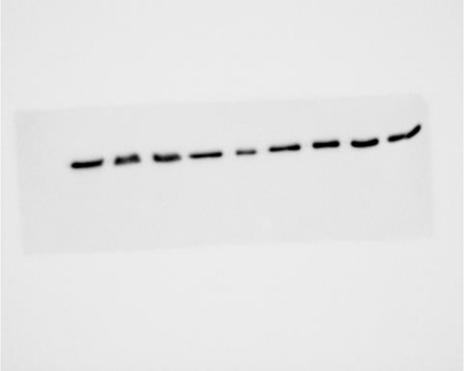

Figure 8. Psychotropic drugs alter autophagy flux through mTOR pathway inhibition. Western blot analysis showing effects on mTOR pathway after 16h treatment with psychotropic drugs on MCF7. Lysates analysed for p-AMPKα T172, AMPK, p-P70S6K T389, P70S6K, P-S6 S235/236, S6, LC3B and GAPDH (**A**). Histogram showing quantification of P70S6K (**B**), S6 (**C**) and AMPK (**D**) phosphorylation normalized on total protein P70S6K and S6. **Raw data of experiment 2**

P-AMPK alpha

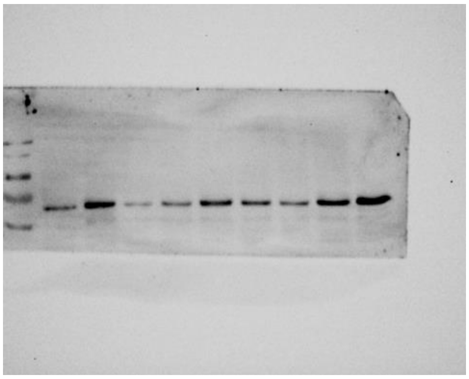

AMPK alpha

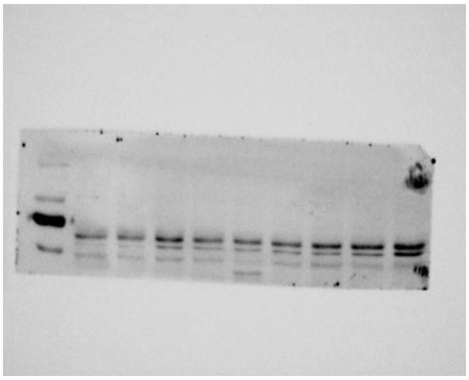

P-P70S6K

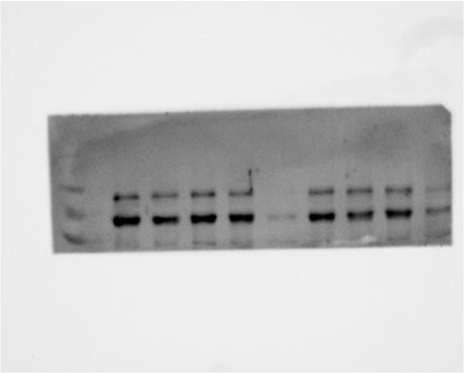

P70S6K

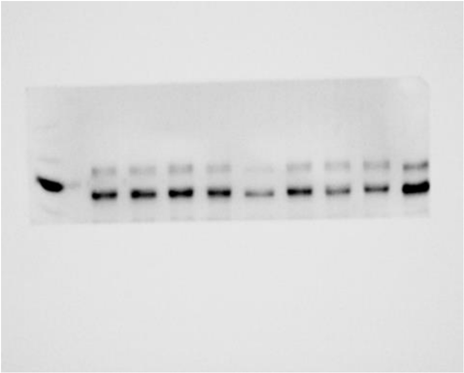

P-S6

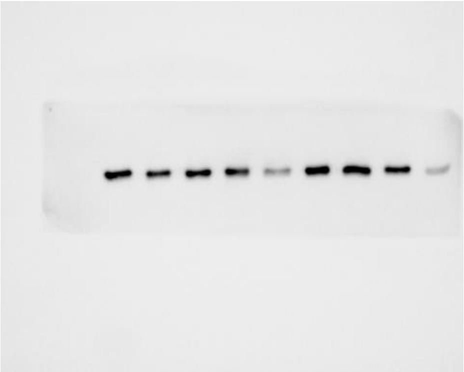

S6

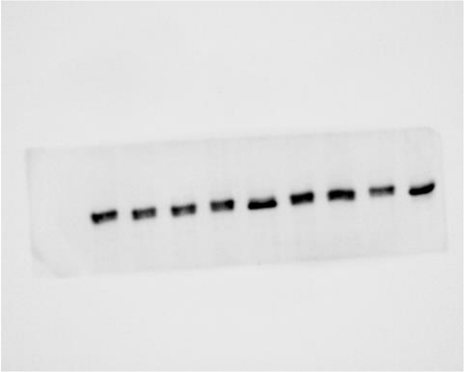

GAPDH

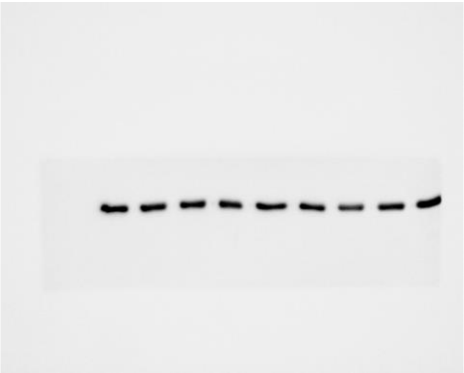

Figure 8. Psychotropic drugs alter autophagy flux through mTOR pathway inhibition. Western blot analysis showing effects on mTOR pathway after 16h treatment with psychotropic drugs on MCF7. Lysates analysed for p-AMPKα T172, AMPK, p-P70S6K T389, P70S6K, P-S6 S235/236, S6 and GAPDH (**A**). Histogram showing quantification of P70S6K (**B**), S6 (**C**) and AMPK (**D)** phosphorylation normalized on total protein P70S6K and S6. **Raw data of experiment 3**

LC3B- DMSO/spiperone/ebastine

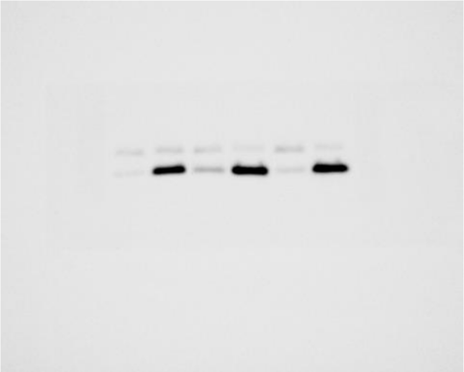

GAPDH- DMSO/spiperone/ebastine

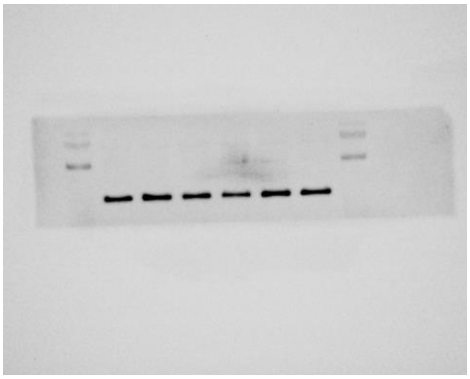

LC3B- pimozide/penfluridol/fluspirilene

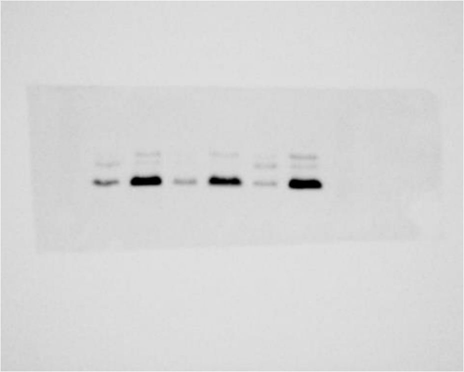

GAPDH- pimozide/penfluridol/fluspirilene

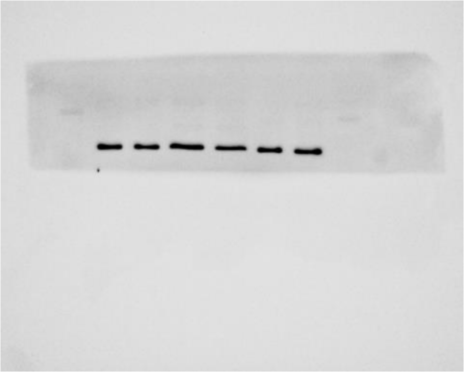

LC3B- fluoxetine/nefazodone/serum starved

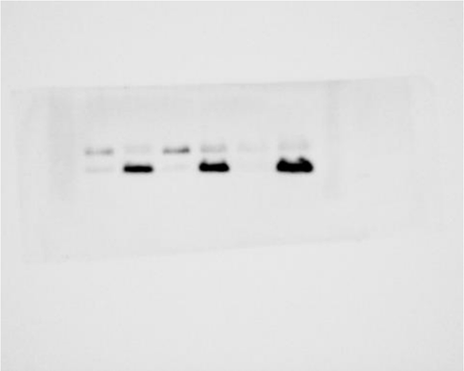

GAPDH- fluoxetine/nefazodone/serum starved

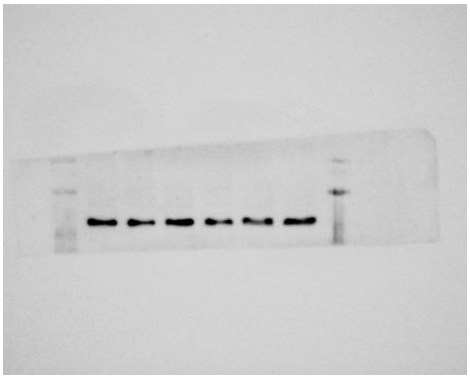

Figure 8. Psychotropic drugs alter autophagy flux through mTOR pathway inhibition. Western blot analysis showing effects on mTOR pathway after 16h treatment with psychotropic drugs on MCF7. WB showing the relative expression of LC3B II/I upon chloroquine treatment **Raw data of experiment 1**

LC3B- DMSO/spiperone/ebastine

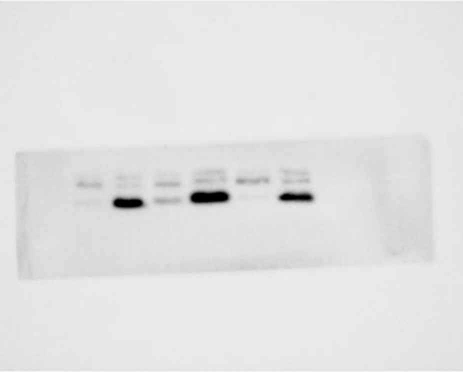

GAPDH- DMSO/spiperone/ebastine

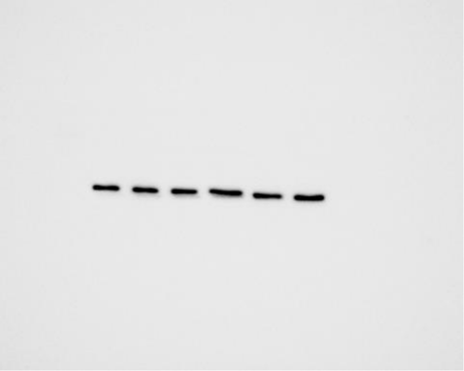

LC3B- pimozide/penfluridol/fluspirilene

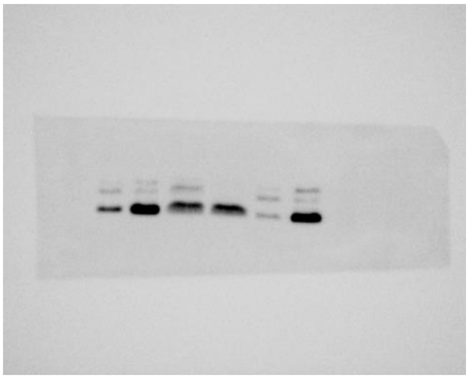

GAPDH- pimozide/penfluridol/fluspirilene

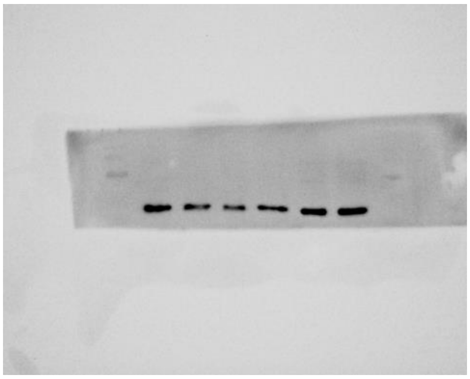

LC3B- fluoxetine/nefazodone/serum starved

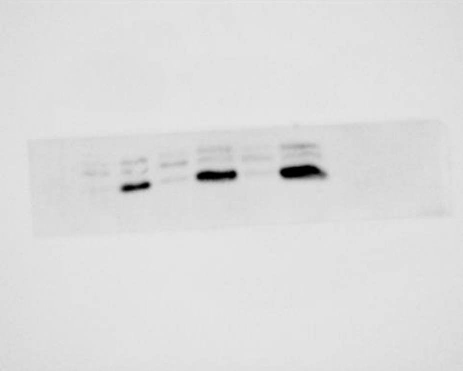

GAPDH- fluoxetine/nefazodone/serum starved

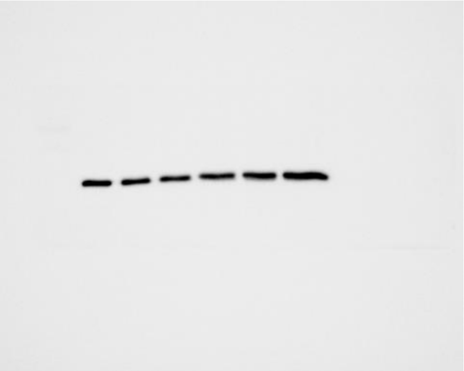

Figure 8. Psychotropic drugs alter autophagy flux through mTOR pathway inhibition. Western blot analysis showing effects on mTOR pathway after 16h treatment with psychotropic drugs on MCF7. WB showing the relative expression of LC3B II/I upon chloroquine treatment **Raw data of experiment 2**

LC3B- DMSO/spiperone/ebastine

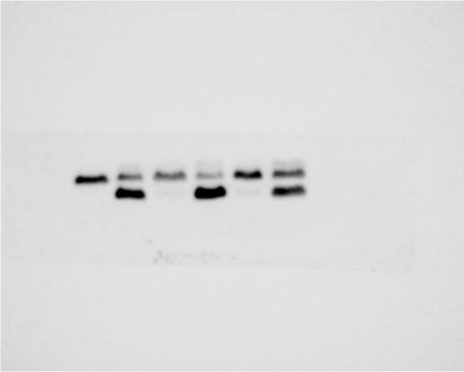

GAPDH- DMSO/spiperone/ebastine

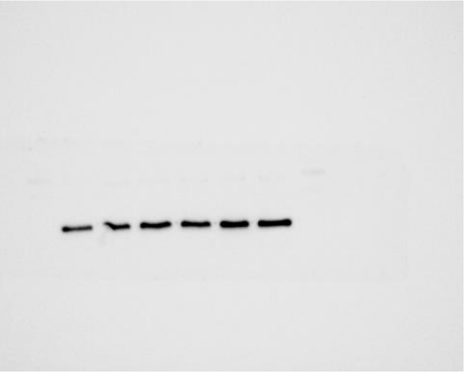

LC3B- pimozide/penfluridol/fluspirilene

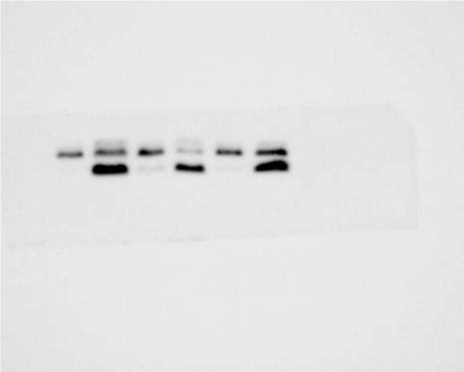

GAPDH- pimozide/penfluridol/fluspirilene

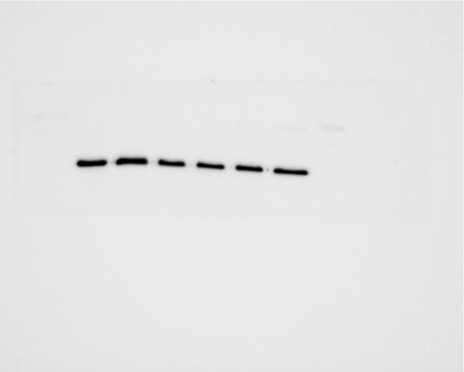

LC3B- fluoxetine/nefazodone/serum starved

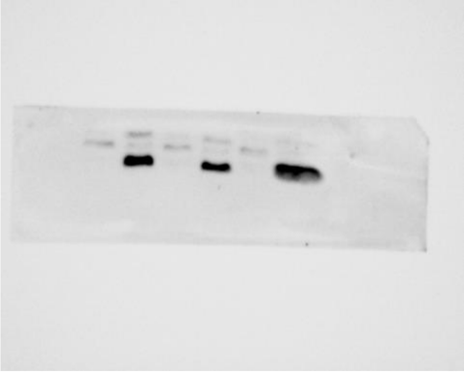

GAPDH- fluoxetine/nefazodone/serum starved

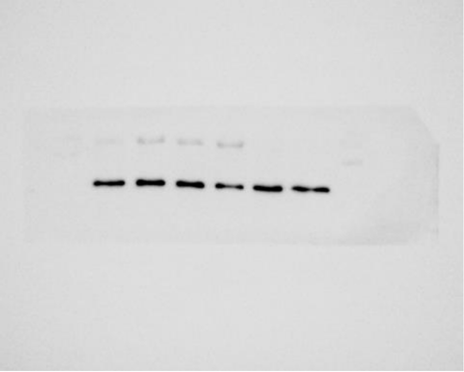

Figure 8. Psychotropic drugs alter autophagy flux through mTOR pathway inhibition. Western blot analysis showing effects on mTOR pathway after 16h treatment with psychotropic drugs on MCF7. WB showing the relative expression of LC3B II/I upon chloroquine treatment **Raw data of experiment 3**

LC3B- experiment 1

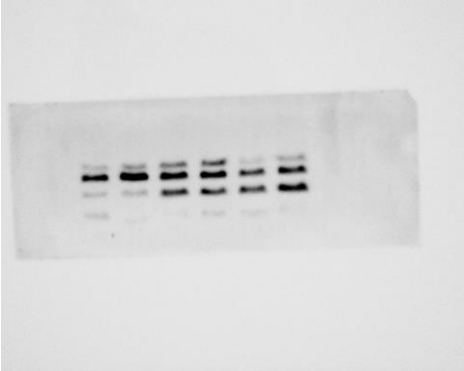

GAPDH- experiment 1

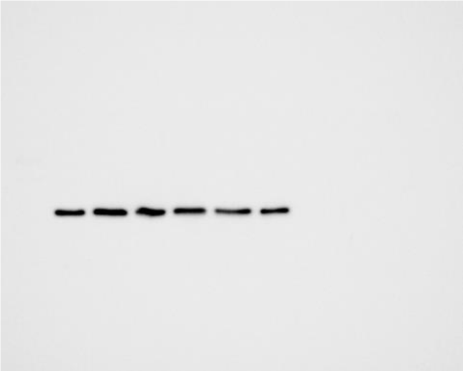

LC3B- experiment 2

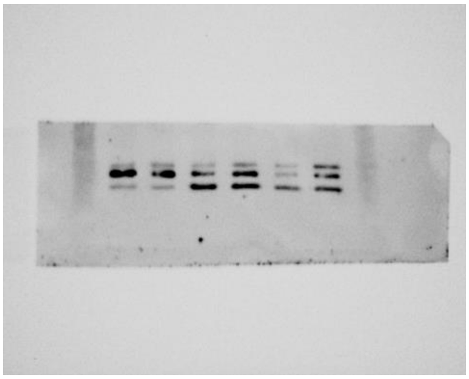

GAPDH- experiment 2

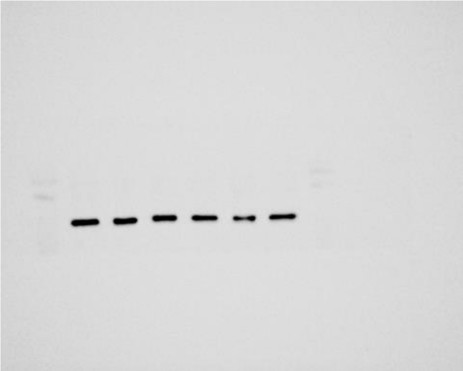

LC3B- experiment 3

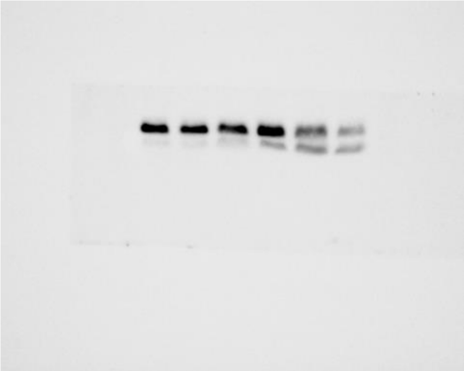

GAPDH- experiment 3

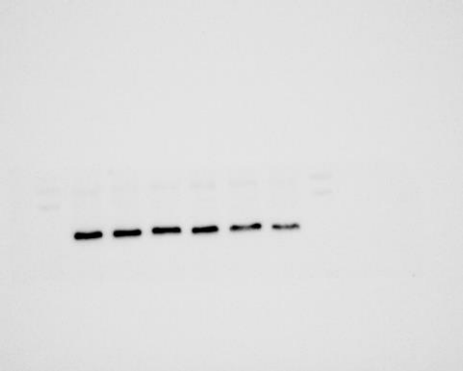

Supplementary Figure 9. 3-MA does not induce LC3B expression upon psychotropic drugs stimulation. Western blot analysis showing effects on autophagic pathway after 16h treatment with spiperone and penfluridol plus 3-MA on MCF7. Raw data of experiment 1
